# The MoSeS dynamic omnigami paradigm for smart shape and composition programmable 2D materials

**DOI:** 10.1038/s41467-019-12945-5

**Published:** 2019-11-15

**Authors:** Joel Berry, Simeon Ristić, Songsong Zhou, Jiwoong Park, David J. Srolovitz

**Affiliations:** 1 Department of Materials Science and Engineering, University of Pennsylvania, Philadelphia, PA 19104 USA; 2 Materials Science Division, Lawrence Livermore National Laboratory, Livermore, CA 94550 USA; 3 Department of Chemistry, Institute for Molecular Engineering, James Franck Institute, University of Chicago, Chicago, IL USA; 4 Department of Mechanical Engineering and Applied Mechanics, University of Pennsylvania, Philadelphia, PA 19104 USA; 5 Department of Materials Science and Engineering, City University of Hong Kong, Hong Kong, SAR P. R. China

**Keywords:** Mechanical properties, Two-dimensional materials, Coarse-grained models, Electronic properties and materials

## Abstract

The properties of 2D materials can be broadly tuned through alloying and phase and strain engineering. Shape programmable materials offer tremendous functionality, but sub-micron objects are typically unachievable with conventional thin films. Here we propose a new approach, combining phase/strain engineering with shape programming, to form 3D objects by patterned alloying of 2D transition metal dichalcogenide (TMD) monolayers. Conjugately, monolayers can be compositionally patterned using non-flat substrates. For concreteness, we focus on the TMD alloy MoSe${}_{2c}$S${}_{2(1-c)}$; i.e., MoSeS. These 2D materials down-scale shape/composition programming to nanoscale objects/patterns, provide control of both bending and stretching deformations, are reversibly actuatable with electric fields, and possess the extraordinary and diverse properties of TMDs. Utilizing a first principles-informed continuum model, we demonstrate how a variety of shapes/composition patterns can be programmed and reversibly modulated across length scales. The vast space of possible designs and scales enables novel material properties and thus new applications spanning flexible electronics/optics, catalysis, responsive coatings, and soft robotics.

## Abstract (web summary)

Current interest in tuning optoelectronic properties of two-dimensional materials focuses on phase and strain engineering. Here the authors propose a novel approach to achieve nanoscale composition/strain patterns and 3D objects with tailored properties using 2D transition metal dicalchogenide alloys.

## Introduction

Shape-programmable materials^1–3^ utilize the large deformations achievable with modest internal strains in thin sheets. The ability to spatially pattern internal strains provides a powerful means to controllably transform 2D sheets into 3D objects. Many biological systems exploit these underlying physical principles^4–6^. Synthetic analogs are employed in soft robotics^7^, drug delivery^8^, biomedical devices^9^, and biomimetic materials^10^. Shape-programmable materials can be engineered to allow dynamic and reversible shape changes (and become smart or 4D materials) by incorporating stimuli-responsive components.

Geometrically, deformations of thin sheets can alter mean ($H$) and/or Gauss ($K$) curvature. Mean curvature is developed by, e.g., constructing a bilayer (analogous to a bimetallic strip) in which the two layers are strained in-plane relative to one another. Gauss curvature requires gradients in in-plane strain within the sheet and can produce a much wider variety of shapes. The ability to independently modulate in-plane and bending strains ($K$ and $H$) enriches the palette of possible 3D shapes and increases shape programmability.

Conceptually, strains can be programmed into sheets as smoothly varying fields (e.g., through lateral or vertical composition/misfit gradients)^11–16^, arrays of topological defects^17–22^ (generated, for example, by conformal growth on substrates with nonflat topographies^22–24^), and “quilted” patches/grains of constant internal strain^25^ (generated, for example, by in-plane heterostructure growth^16,26–28^ and localized phase transformations^29–32^). Material type and synthesis/processing conditions dictate which of these are realizable and the length scales of the achievable 3D shapes.

Soft shape programmable materials based on hydrogels^33^, polymers^34^, nematic elastomers^35^, and other shape memory materials^36^ have been extensively studied. Means to control both bending^37–39^ and in-plane deformations^11,33,40^ have been developed, in addition to stimuli-responsive, reversibly actuatable materials^33,41^. However, since film thicknesses are often large (e.g., ${\sim} 10$ µm), 3D objects made from such soft films typically have large characteristic radii of curvature (${\sim} 0.1$ mm).

Atomically thin materials are required for atomic scale programming. To date, 3D feature generation involving inorganic 2D materials^42^, with thicknesses $<$1–10 nm, has largely been explored using thicker composite bilayers or bimetallic strips^43–45^, mechanical self-assembly/buckling-based approaches (e.g., patterned sheets on thicker prestrained deformable substrates^46–49^), and cut-and-deform/kirigami approaches^50,51^. Quasi-2D materials with novel thermal^52,53^, mechanical^54^, optical^55,56^, and electronic^57,58^ properties have thus been produced. However, programmed atomic scale 3D features are not readily realized with such approaches.

Here, we propose and theoretically investigate an approach based on single 2D transition metal dichalcogenide (TMD) alloy monolayers that permits composition programming to achieve nanoscale 3D objects with exquisite shape control through both bending and stretching (which we dub omnigami) and is rapidly and reversibly actuatable with external electric fields. This system also facilitates the conjugate process; using imposed shapes to self-assemble composition patterns during synthesis or postprocessing. The resultant composition patterns imprint shape, which may be retained when the shape control is removed (e.g., TMD annealing on and removal from a nonplanar substrate). We explore the shape and composition programmable features of TMD alloy monolayers by developing a first principles-informed continuum model for their coupled mechanics, alloy thermodynamics, and interaction with electric fields. The fundamental relations between composition and deformation are derived for several simple shapes and patterns. We then numerically demonstrate the ability to program a wide variety composition patterns and 3D shapes, from smoothly curved to purely fold-based origami designs, with a wide variety of strain states, from internally strain-free to patterns with designed residual strains (e.g., to obtain target mechanical or electronic properties). We also demonstrate how the electrically dipolar nature of Janus (see below) TMDs enables dynamic actuation of these self-shaping 2D materials with electric fields for smart or 4D material functionality. Potential applications of patterned, shaped, and responsive TMD monolayers in optoelectronic devices, flexible electronics, catalysis, responsive coatings, and soft robotics are discussed. Demonstrations include designed composition patterns that can be employed in electronic devices with spatially tailored bandgaps, bilayers with programmed twist angles, and sheets with corrugated/compliant, crumpled, or channeled geometries of nearly arbitrary complexity.

## Results

### Physical concept

**Fig. 1:**
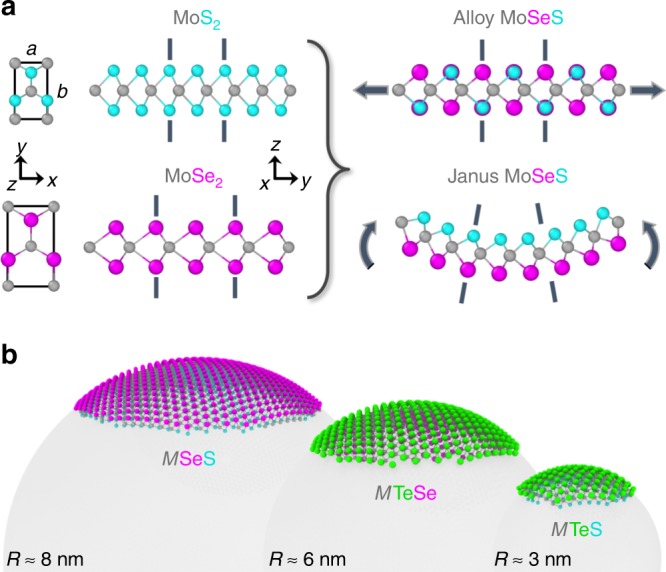
Coupling between composition and deformation in TMD alloy monolayers. **a** The TMD hexagonal crystal structure and illustrations of compositionally generated stretching and bending deformations in MoSeS alloys (the $z$-direction is normal to the TMD monolayer). Metal and chalcogen atoms are shown in gray and cyan/magenta, respectively. The S/Se size difference is amplified for visual clarity. **b** Equilibrium geometries of Janus $M$SeS, $M$TeSe, and $M$TeS patches

TMD monolayers ($M{X}_{2}$) contain three covalently bonded atomic layers; a transition metal atomic layer M ${\rm{\in \{Mo,W\}}}$ sandwiched between chalcogen $X{\rm{\in \{S,Se,Te\}}}$ atomic layers. Here, we consider binary chalcogen alloy monolayers. Since different chalcogens have different atomic sizes, in-plane variations in the average chalcogen concentration $\bar{c}=({c}_{+}+{c}_{-})/2$ (where +/− refers to the upper/lower chalcogen layers) generate variations in in-plane strain (Fig. 1a). Similarly, vertical variations in chalcogen concentration (quantified in terms of Janus degree $J=({c}_{+}-{c}_{-})/2$) generate bending strains (Fig. 1a). A difference in concentrations between the chalcogen layers creates a preferred radius of curvature (bending). The minimum radii of curvature for Janus $M$SeS, $M$TeSe, and $M$TeS are ${R}_{{\rm{c}}}$  $\approx$  $h{a}_{X}/({a}_{X}-{a}_{Y})$  $\approx \ 8$, 6, and 3 nm, respectively ($h$ is the monolayer thickness, ${a}_{X}$ and ${a}_{Y}$ are the lattice constants of $M{X}_{2}$ and $M{Y}_{2}$; see Fig. 1b). Spatially uniform Janus TMD monolayers are novel piezoelectric materials^59–63^. Here, we propose using the natural curvature of Janus TMDs^60^ in shape programming by patterning lateral variations in Janus degree and thus curvature.

The conjugate material programming processes explored here are referred to as shape $\to$ composition programming (patterning topography to program composition patterns) and composition $\to$ shape programming (patterning composition to program non-flat monolayer topographies). These can both be viewed as forward problems; for a given input (shape or composition), determine the output (composition or shape). Or, as inverse/design problems; given a target output (shape or composition), determine the input (composition or shape) required to generate it. Our presentation is framed in terms of the forward problems which provide a simpler framework for conveying the physical principles and methodology behind dynamic MoSeS omnigami. We return to the inverse/design problems in the Discussion.

### First principles-informed continuum model

We consider a single alloy TMD monolayer $M{X}_{2\bar{c}}{Y}_{2(1-\bar{c})}$, where $\bar{c}$ is the composition of chalcogen species $X$ averaged over both chalcogen layers. The monolayer free energy functional is decomposed as

1 $${\mathcal{F}}={\mathcal{F}}_{\rm{elastic}}+{\mathcal{F}}_{{c}_{+}}+{\mathcal{F}}_{{c}_{-}}+{\mathcal{F}}_{\rm{electric}},$$

where ${\mathcal{F}}_{{\rm{elastic}}}$ is the elastic energy of the heterogeneously alloyed monolayer, ${\mathcal{F}}_{{c}_{+}}+{\mathcal{F}}_{{c}_{-}}$ is the compositional free energy of the upper and lower flat chalcogen layers in the absence of an electric field, and ${\mathcal{F}}_{{\rm{electric}}}$ is the electrostatic energy of the heterogeneously alloyed (dipolar/Janus) monolayer in an applied electric field.

### Elastic energy

The mechanics of the monolayer is described using the Föppl-von Kármán thin plate theory extended to include heterogeneous bending eigenstrain fields associated with the Janus nature of the monolayers and stretching eigenstrain fields associated with in-plane misfit from variations in the composition (averaged over the two chalcogen layers at each position in the monolayer). The elastic free energy functional has contributions from the in-plane strain $\epsilon$ and the mean out-of-plane displacements $w$

2 $${\mathcal{F}}_{{\rm{elastic}}}={\mathcal{F}}_{\epsilon }+{\mathcal{F}}_{w}$$

3 $${\mathcal{F}}_{\epsilon }=\frac{\tilde{h}{\lambda }_{ijkl}}{2}{\int }_{A}({\epsilon }_{ij}-{\epsilon }_{ij}^{* })({\epsilon }_{kl}-{\epsilon }_{kl}^{* })dA$$

4 $${\mathcal{F}}_{w}=\frac{{\tilde{h}}^{3}{\lambda }_{ijkl}}{24}{\int }_{A}({w}_{,ij}-{w}_{ij}^{* })({w}_{,kl}-{w}_{kl}^{* })dA,$$

where *a* “,” indicates partial differentiation with respect to the subsequent variable(s). Here, $$\tilde{h}$$ is the effective elastic plate thickness, $${\lambda }_{ijkl}$$ is the elastic stiffness tensor, $$A$$ is the area of the monolayer, $${\epsilon }_{ij}={\bar{\epsilon }}_{ij}+({u}_{i,j}+{u}_{j,i}+{w}_{,i}{w}_{,j})/2$$ is the local elastic stretching deformation tensor associated with (small) in-plane displacements $${u}_{i}$$ and (potentially large) out-of-plane displacements $$w$$, $${\bar{\epsilon }}_{ij}$$ is a (macroscopic) applied strain and $$i,j,k,l\in \{x,y\}$$. The in-plane misfit/eigenstrain is set by the average local composition within the chalcogen layers (i.e., solutes are point sources of dilatation) as $${\epsilon }_{ij}^{* }={\delta }_{ij}\check{\epsilon }\delta \bar{c}/(1+\check{\epsilon }{\bar{c}}_{0})$$, where $$\delta \bar{c}=\bar{c}-{\bar{c}}_{0}=\frac{1}{2}({c}_{+}+{c}_{-})-{\bar{c}}_{0}$$, $${\bar{c}}_{0}$$ is the average local composition of the eigenstrain reference state (the zero eigenstrain state), $$\check{\epsilon }=$$$$({a}_{X}-{a}_{Y})/{a}_{X}$$ is the maximum eigenstrain, and $${\delta }_{ij}$$ is the Kronecker delta. $${w}_{ij}^{* }=2J\check{\epsilon }{\delta }_{ij}/h(1+\check{\epsilon }\bar{c})$$ is the local spontaneous curvature tensor for a given local composition difference between the two chalcogen layers, where $$h$$ is the geometric thickness of the monolayer (vertical $$z$$ distance between chalcogen atomic layer mid-planes). The interatomic layer thickness $$h$$ is a better indicator for the compositional effect on bending eigenstrain than the (distinct, though related) $$\tilde{h}$$, proportional to the square root of the ratio between the bending and stretching stiffnesses. From these definitions of deformation, elastic strain is $${\epsilon }_{ij}-{\epsilon }_{ij}^{* }$$.

Since hexagonal (H phase) TMD monolayers are elastically isotropic, Eqs. (3) and (4) can be rewritten with the plane strain ${\lambda }_{ijkl}=E[2\nu {\delta }_{ij}{\delta }_{kl}/(1-\nu )+{\delta }_{ik}{\delta }_{jl}+{\delta }_{il}{\delta }_{jk}]/2(1+\nu )$, where $E$ and $\nu$ are the in-plane Young’s modulus and Poisson’s ratio, respectively.

### Compositional energy

The alloy free energies of TMD monolayers with alloyed metal^64^/chalcogen^65^ layer(s) have previously been calculated within a regular solution model. Here, we generalize the regular solution model for each chalcogen layer in MoSeS-type alloys by inclusion of coupling between layers

5 $$\begin{array}{ll}{\mathcal{F}}\!\!_{{c}\!_{\pm }}=&\frac{1}{2}\int_{A}\left\{{\tilde{k}}_{{\rm{B}}}T\left[{c}\!_{\pm }\mathrm{ln}({c}\!_{\pm })+(1-{c}\!_{\pm })\mathrm{ln}(1-{c}\!_{\pm })\right]\right.\\ & +\, {f}_{{\rm{mix}}}({c}\!_{\pm })+{\gamma }^{2}| \nabla {c}\!_{\pm }{| }^{2}+\frac{\Lambda }{2}{({c}\!_{\pm }-{c}_{\mp })}^{2}-{\mu }_{0}{c}\!_{\pm }\left.\right\}dA,\end{array}$$

where ${\tilde{k}}_{{\rm{B}}}T$ is the usual thermal energy, ${f}\!_{{\rm{mix}}}({c}\!_{\pm })$ is the enthalpy of mixing within a given chalcogen layer, $\gamma$ scales the interface energy between regions of different composition in-plane, and the constant $\Lambda$ characterizes the strength of the local interaction between the two chalcogen layers. ${\mu }_{0}$ is a chemical potential that controls the relative $X$–$Y$ composition (e.g., set by the partial pressure of the different species in the monolayer environment).

### Electrical potential energy

Since the electronegativity of species $$X$$ and $$Y$$ are different, a Janus region of a monolayer is electrically polar, with local dipole moment density $${\bf{p}}$$$$\ =2{p}_{0}J$$ $$\hat{{\bf{n}}}$$, where $${p}_{0}$$ is the saturated dipole moment density ($$J=1/2$$) and $$\hat{{\bf{n}}}$$ is the local normal vector of the monolayer. The interaction energy between dipoles and with an applied electric field $${{\bf{E}}}_{{\rm{A}}}$$ is

6 $${\mathcal{F}}\!_{{\rm{electric}}}=\int {\bf{p}}({\bf{r}})\cdot \left(\frac{1}{4\pi {\epsilon }_{0}}\nabla \int \frac{\nabla \cdot {\bf{p}}({\bf{r}}^{\prime})}{| {\bf{r}}-{\bf{r}}^{\prime} | }{\bf{dr}}^{\prime} +{{\bf{E}}}_{{\rm{A}}}({\bf{r}})\right){\bf{dr}}.$$

The first term (dipole–dipole interaction) is higher order than the second term and is typically negligible compared to compositional and elastic energies in MoSeS (see Methods, Supplementary Fig. 1, and Supplementary Note 1 for details); hence, it is omitted below.

### Physical parameters

All physical parameters describing MoSeS in $$\mathcal{F}$$ have been computed from first principles calculations and are listed in Supplementary Table 1. The parameters $${a}_{X}$$, $${a}_{Y}$$, $$h$$, $$\tilde{h}$$, $$E$$, $$\nu$$, $$\kappa$$, and $${p}_{0}$$ were determined from previous DFT calculations^60,65–67^; alleviating the need to determine continuum plate theory parameters based on assumptions such as, e.g., effective plate thickness. Comparisons of plate theory with DFT calculations show that the two are in reasonable agreement (e.g., for the stiffness $$\kappa$$)^67^. Thus, the primary assumption in the continuum plate theory is to only include the lowest order deformation terms in the elastic energy, consistent with the small strains encountered here ($${\ll} 0.1$$). Since the elastic moduli of MoS$${}_{2}$$ and MoSe$${}_{2}$$ are similar (15–20% variation), we neglect the elastic inhomogeneity associated with compositional variations within the monolayer and use overall composition-weighted averages of $$E$$, $$\nu$$, $$\kappa$$, and $$h$$ for the entire monolayer. The ($$T=0$$ K) enthalpy of mixing was parameterized as an asymmetric regular solution, $${f}\!_{{\rm{mix}}}({c}\!_{\pm })=\chi {c}\!_{\pm }(1-{c}\!_{\pm })+{c}\!_{\pm }$$$${U}_{X}$$$$+(1-{c}\!_{\pm })$$$${U}_{Y}$$. The regular solution constants ($$\chi$$, $${U}_{X}$$, $${U}_{Y}$$) and $$\Lambda$$ (characterizing compositional interactions between chalcogen layers) were computed from first principles as described in Methods. The constant $$\gamma$$ in Eq. (5) characterizes interfaces between domains of different composition—not encountered in these TMD solid solutions. Here, it should be viewed as a regularization parameter that prevents excessively sharp concentration gradients from forming; it is chosen large enough to facilitate numerical stability but small enough to have a negligible effect on composition patterns.

### Simulation schemes

Shape ${\to}$ composition programming was simulated by numerically evolving the ${c}_{+}$ and ${c}_{-}$ fields to the state of minimum ${\mathcal{F}}$ at fixed ${u}_{i}$ and $w$. This constraint on ${u}_{i}$ and $w$ corresponds to (nonslipping) adhesion between the monolayer and a rigid substrate. Imperfect adhesion/conformation and/or slipping will result in somewhat less compositional variation than predicted here.

Composition $\to$ shape programming was simulated by evolving the elastic state fields (${u}_{i},w$) to the state of minimum ${\mathcal{F}}$ at fixed ${c}\!_{\pm }$. The equilibrium ${u}_{i}$ were calculated analytically. $w$ was determined by numerical solution of a damped wave equation that approximates flexural acoustic phonon dynamics (vibrational effects are not considered in our free energy ${\mathcal{F}}$). Imposing no constraints on ${u}_{i}$ and $w$ corresponds to a freely-suspended sheet. However, the simulations reported here were performed on a square or rectangular unit cell with periodic boundary conditions to represent large sheets within a manageable computational domain. See Methods for further details.

### Shape $\to$ composition programming

In the following subsections we examine how patterned monolayer shape (topography) can be used to generate composition patterns.

### Programmed Janus monolayers

Consider first the simple case of a small patch of the surface of a sphere of radius $R$. For a sufficiently small patch, the stretching energy makes higher order contributions than the first-order bending energy and can be neglected. The composition profile that minimizes the bending energy ${{\mathcal{F}}}\!_{w}$ (in the absence of an applied electric field) is a homogeneous Janus monolayer of degree $J\ \approx \ h/2R\check{\epsilon }$. For example, a patch of $R=8$, 80, and 800 nm in MoSeS corresponds to $J\ \approx \ 0.5$, 0.05, 0.005.

However, the total energy also includes compositional contributions. Minimization of the bending and compositional energies with respect to $J$ for a spherical patch of radius $R$ (with mixing entropy expanded to third order about $J=0$) gives

7 $$J\approx \frac{h}{2R\check{\epsilon }\left(1+{\Delta }_{w}\right)}$$

where

8 $${\Delta }_{w}=\frac{2\Lambda -\chi +{\tilde{k}}_{{\rm{B}}}T/2\bar{c}(1-\bar{c})}{4\kappa (1+\nu ){\check{\epsilon }}^{2}/{h}^{2}}.$$

**Fig. 2:**
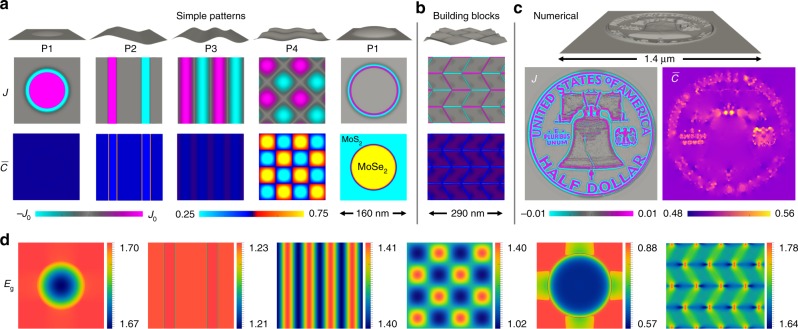
Shape $\to$ composition programming with MoSeS monolayers. Simulated equilibrium composition patterns ($J$ and $\bar{c}$) generated at $1023$ K by the topographies shown at the top of each column. **a** Simple patterns (see the first column of Table 1 for expressions), **b** A Miura-ori pattern that employs P2 as a building-block structure, **c** An elaborate pattern obtained *via* our numerical programming approach. In **a** and **b**, the $J$ scale is set to the predicted maximum ${J}_{0}$ of each topography (second column of Table 1), while the $\bar{c}$ scale varies from $0.25$ to $0.75$. The last column in **a** shows a large amplitude P1 template (large stretching) that generates an in-plane MoS${}_{2}$/MoSe${}_{2}$ heterostructure. Regions in gray in the Janus degree maps represent $J=0$. **d** Maps of electronic bandgap ${E}_{{\rm{g}}}$ (in eV) for the five patterns shown in **a** and the Miura-ori pattern in **b**, based on DFT calculations of the composition and strain dependent bandgaps in refs. ^60,68,69^. See Supplementary Note 2 for more details

**Fig. 3:**
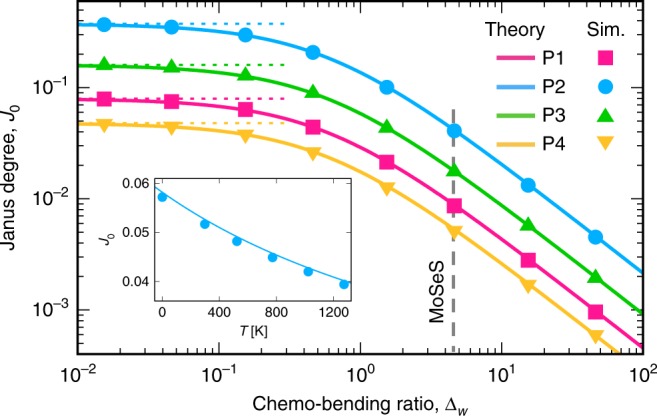
Effect of compositional thermodynamics on shape $\to$ composition programming. Simulation results validating analytic predictions for bending-dominated monolayers are also shown. Solid and dashed lines are the expressions in the second column (Composition $J$) of Table 1 and the ${{\mathcal{F}}}_{{c}\!_{\pm }}=0$ limit (${\Delta }_{w}\to 0$), respectively, and points are simulation results obtained at equal Se and S concentrations ($\bar{c}=1/2$). ${J}_{0} \sim 1/{\Delta }_{w}$ for ${\Delta }_{w}\gg 1$. Inset: ${J}_{0}$ vs. $T$ for P2 (a 1D bend)

The chemo-bending ratio ${\Delta }_{w}$, a ratio of chemical to elastic bending energies, quantifies how compositional energy suppresses/enhances inhomogeneous spatial Janus patterns (depending on $\chi$ and $\Lambda$) relative to the ${{\mathcal{F}}}\!_{{c}\!_{\pm }}=0$ prediction, $J=h/2R\check{\epsilon }$. For MoSeS, $1+{\Delta }_{w}\ \approx \ 5.6$ at $T=0$ K and $7.6$ at a reasonable growth/annealing temperature $T=1023$ K. Therefore, generating a Janus MoSeS spherical patch with $J=0.5$, 0.05, 0.005 at $T=1023$ K requires monolayer patches with radii of curvature $R\ \approx \ 1$, 10, and 100 nm, respectively. Simulations including all terms in Eqs. (2) and (5) confirm the predictions of Eq. (7) as shown in Figs. 2a and 3.

Eq. (7) demonstrates that the ease with which large compositional variations can be programmed into a monolayer depends on the competition between compositional and elastic energies. While composition rearrangement relaxes the strain energy associated with monolayer topography, the resulting inhomogeneous compositions have relative compositional energy costs proportional to ${\Delta }_{w}$. Large compositional variations are most readily programmed in monolayers with small ${\Delta }_{w}$, but similar results can be obtained in materials with relatively large ${\Delta }_{w}$, such as MoSeS, provided that sufficiently large topography amplitudes (large curvature in this case) can be realized. We now examine this and related issues for a series of different compositional patterns.

### Programmed alloy enrichment

We next consider uniform changes in alloy concentration by exchange of species with a reservoir; e.g., by annealing in atmospheres with different chalcogen chemical potentials ${\mu }_{0}$ (partial pressures) and/or under homogeneous isotropic in-plane strain $\bar{\epsilon }={\bar{\epsilon }}_{xx}={\bar{\epsilon }}_{yy}$. Minimization of the stretching and compositional energies with respect to $\bar{c}$ for a flat monolayer under tension $\bar{\epsilon }$ (with mixing entropy expanded to second order about $\bar{c}=1/2$) gives a linear relation between $\bar{c}$ and strain,

9 $$\bar{c}\approx \frac{\bar{\epsilon }}{\beta (1+{\Delta }_{\epsilon })}+{\bar{c}}_{{\rm{eq}}},$$

where

10 $${\Delta }_{\epsilon }=\frac{-\chi +2{\tilde{k}}_{{\rm{B}}}T}{2\alpha {\beta }^{2}}$$

and

11 $${\bar{c}}_{{\rm{eq}}}=\frac{\chi +{U}_{X}-{U}_{Y}-{\mu }_{0}-4\alpha {\beta }^{2}{\bar{c}}_{0}-2{\tilde{k}}_{{\rm{B}}}T}{2(\chi -2\alpha {\beta }^{2}-2{\tilde{k}}_{{\rm{B}}}T)}.$$

${\Delta }_{\epsilon }$ is the chemo-stretching ratio, ${\bar{c}}_{{\rm{eq}}}$ is the equilibrium composition at zero strain, $\alpha =\tilde{h}E/2(1-\nu )$, and $\beta =\check{\epsilon }/(1+\check{\epsilon }{\bar{c}}_{0})$.

Analogous to ${\Delta }_{w}$, ${\Delta }_{\epsilon }$ quantifies the degree to which compositional thermodynamics alters the ${{\mathcal{F}}}\!_{{c}\!_{\pm }}=0$ result $\bar{c}\ \approx \ \bar{\epsilon }/\beta +{\bar{c}}_{{\rm{eq}}}$. For MoSeS, ${\Delta }_{\epsilon }\ \approx \ 1.2$ at $T=0$ K and ${\Delta }_{\epsilon }\ \approx \ 2.6$ at $T=1023$ K; this indicates that the compositional energy has a significantly weaker effect on shape-programmed alloy composition (dominated by stretching) than on shape-programmed Janus composition (dominated by bending, Eq. (7)). This difference is associated with the vast difference in the stretching and bending stiffnesses of TMD monolayers.

### Simple patterns

We now focus on some simple monolayer topographies $w(x,y)$ and the Janus composition patterns $J(x,y)$ they produce. Results for various topographies, obtained by minimizing ${{\mathcal{F}}}\!_{w}+{{\mathcal{F}}}\!_{{c}\!_{\pm }}$ with respect to $J(x,y)$ at fixed $w(x,y)$, are given in Table 1. To retain the analytical nature of these predictions, we ignore the relatively small contributions of the in-plane strain associated with the substrate topography. This approximation is typically valid for monolayer islands with free edges and/or slowly varying topographies.

**Table 1:** Equilibrium mappings for shape $\to$ composition programming and composition $\to$ shape programming

Shape $\to$ Composition			Composition $\to$ Shape			
Shape $w(x,y)$	$\longrightarrow$	Composition $J(x,y)$	Composition $J(x,y)$	$\longrightarrow$	Shape Amplitude	
		$w$ dominant			$w$ dominant	$\epsilon$ dominant
P1: $\sqrt{{R}^{2}-{x}^{2}-{y}^{2}}$		$\frac{h}{2R\check{\epsilon }\left(1 + {\Delta }_{w}\right)}$	${J}_{0}H({r}_{0}-\sqrt{{x}^{2}+{y}^{2}})$		$\frac{{r}_{0}^{2}}{R}=\frac{2\check{\epsilon }{J}_{0}{r}_{0}^{2}}{h}$	${\left(\frac{9h{J}_{0}{r}_{0}^{2}}{2{E}_{\epsilon }}\right)}^{1/3}$
P2: $\frac{1}{2}B{x}^{2}$		$\frac{hB}{4\check{\epsilon }\left(1 + {\Delta }_{w}\right)}$	${J}_{0}{\rm{rect}}(x/L)$		$B{L}^{2}=\frac{\tilde{\nu }\check{\epsilon }{J}_{0}{L}^{2}}{h}$	${\left(\frac{16h{J}_{0}{L}^{3}}{{E}_{\epsilon }d}\right)}^{1/3}$
P3: $A\sin (kx)$		$\frac{-h{k}^{2}w(x)}{4\check{\epsilon }\left(1 + {\Delta }_{w}\right)}$	${J}_{0}\sin (kx)$		$A=\frac{\tilde{\nu }\check{\epsilon }{J}_{0}}{{k}^{2}h}$	${\left(\frac{h{J}_{0}{\tilde{\nu }}^{2}}{2{E}_{\epsilon }{k}^{2}}\right)}^{1/3}$
P4: $\frac{A}{2}\left[\sin (kx)+\sin (ky)\right]$		$\frac{-h{k}^{2}w(x,y)}{4\check{\epsilon }\left(1 + {\Delta }_{w}\right)}$	$\frac{{J}_{0}}{2}\left[\sin (kx)+\sin (ky)\right]$		$A=\frac{\tilde{\nu }\check{\epsilon }{J}_{0}}{{k}^{2}h}$	${\left(\frac{h{J}_{0}\tilde{\nu }}{2{E}_{\epsilon }{k}^{2}}\right)}^{1/3}$
P5: $C\sqrt{\frac{\pi }{2}}\, x{\rm{erf}}\left(\frac{\sqrt{2}x}{2\sigma }\right)+C\sigma {e}^{\frac{-{x}^{2}}{2{\sigma }^{2}}}$		$\frac{hC{e}^{-{x}^{2}/2{\sigma }^{2}}}{4\sigma \check{\epsilon }\left(1 + {\Delta }_{w}\right)}$	${J}_{0}{e}^{-{x}^{2}/2{\sigma }^{2}}$		$C\sigma =\frac{\tilde{\nu }\check{\epsilon }{J}_{0}{\sigma }^{2}}{h}$	${\left(\frac{4h{J}_{0}{\sigma }^{2}}{{\pi }^{3/2}{E}_{\epsilon }}\right)}^{1/3}$
**Strain** ${\bar{\epsilon }}_{ij}$	$\longrightarrow$	**Composition** $\bar{c}$	**Composition** $\bar{c}$	$\longrightarrow$	**Strain** $\bar{\epsilon }$	–
$\bar{\epsilon }={\bar{\epsilon }}_{xx}={\bar{\epsilon }}_{yy}$		$\frac{\bar{\epsilon }}{\beta (1 + {\Delta }_{\epsilon })}+{\bar{c}}_{{\rm{eq}}}$	$\bar{c}$		$\beta (\bar{c}-{\bar{c}}_{0})$	–

Analytical mappings in limits where the elastic energy is dominated by bending or stretching (i.e., $w$ or $\epsilon$ dominant). All shapes $w$ refer back to the equations for the patterns P1-P4 (Fig. 2a) on the left side of the table (**Shape** column) and their amplitude parameters ($1/R$, $B$, $A$, and $C\sigma$). ${E}_{\epsilon }=E{h}^{2}\tilde{h}/2\kappa (1-{\nu }^{2})\tilde{\nu }\check{\epsilon }$, $\tilde{\nu }=2(1+\nu )$, and $d$ is the in-plane distance between quadratic folds in P2

The results demonstrate that Janus patterns can be directly obtained by annealing on substrates with patterned topographies and that compositional energy introduces a factor of ${(1+{\Delta }_{w})}^{-1}$ into the programmed Janus degree for each topography. For MoSeS monolayers, this effect suppresses $J$ by a factor of $\sim$5.6 to 8. Simulation results are consistent with these predictions, as shown in Fig. 3 for spherical cap (P1), 1D bend (P2), 1D sine (P3), and 2D sine (P4) patterns. The simulation configurations shown in Fig. 2a demonstrate further how bending-dominated topographies (e.g., small amplitude as in P1-left or unidirectional patterns as in P2 and P3) generate weak spatial $\bar{c}$ variations, while topographies that impose significant stretching (e.g., large amplitude as in P1-right or bidirectional patterns as in P4) generate pronounced spatial $\bar{c}$ variations. More precisely, we find that $\bar{c}$ pattern magnitude $\sim {A}^{2}$ while $J$ pattern magnitude $\sim A$ (Table 1), which leads to the observed $J$ prominence at small $A$ and $\bar{c}$ prominence at large $A$. It is also seen that $\bar{c}$ pattern periods are half that of the corresponding $J$ pattern. This is associated with the different symmetries of the in-plane strains and curvatures with respect to $w$; $\bar{c} \sim {\epsilon }_{ij} \sim {w}_{,i}{w}_{,j}$ while $J \sim {w}_{,ij}$. For example, for P3 ${w}_{,i}{w}_{,j} \sim {\cos }^{2}(kx) \sim \cos (2kx)$, while ${w}_{,ij} \sim \sin (kx)$.

### Complex patterns and scale dependence

More complex composition patterns can be constructed by combining these relatively simple patterning elements as building blocks. One example is the Miura-ori paper folding pattern shown in Fig. 2b. For patterns less amenable to analytic approximations, numerical simulations provide the necessary deformation-composition mappings. Our numerical approach to forward shape $\to$ composition programming simply requires equilibrating the composition of a monolayer while it is held in contact with the shape-patterned substrate template.

Figure 2c shows a complex composition pattern (the reverse of the US Franklin half dollar) programmed into a $\bar{c}=1/2$ MoSeS monolayer using this approach. Distinct $J$ and $\bar{c}$ patterns are obtained from this topography, with $J$ localizing to the most highly curved regions and $\bar{c}$ localizing to the most highly stretched or compressed regions.

While the spatial dimensions of programmed composition patterns can be varied from the nano- to macro-scale, not all terms in the total free energy $\mathcal{F}$ scale with spatial dimensions in the same manner. Topographies scaled equally in each dimension by a dimensionless scaling factor $\mathcal{L}$, $({L}_{x},{L}_{y},w)\to ({L}_{x},{L}_{y},w)\mathcal{L}$, produce nearly self-similar $\bar{c}$ patterns while the amplitude of the $J$ pattern scales ${\sim} \, 1/\mathcal{L}$. This may be traced to the fact that $\bar{c}$ redistribution is driven by in-plane strains ${\epsilon }_{ij} \sim {w}_{,i}{w}_{,j}$ which are invariant under such a transformation, while $J$ redistribution is driven by curvature ${w}_{,ij}$ which scales as $1/\mathcal{L}$. As the overall size scale of a pattern $\mathcal{L}$ decreases, ${\epsilon }_{ij}^{* } \sim \delta \bar{c}$ remains constant while ${w}_{ij}^{* } \sim J$ must increase by the same proportion to satisfy the increasing curvature ${w}_{,ij}$. Conversely, the amplitude of the $J$ pattern is invariant under curvature-conserving topography transformations, e.g., $({L}_{x},{L}_{y},w)\to ({L}_{x},{L}_{y},w{\mathcal{L}})\mathcal{L}$, while the amplitude of the $\bar{c}$ pattern $\sim \mathcal{L}$. It is therefore possible (within the limit of strains that may be produced in a particular alloy system) to vary the spatial dimensions of composition patterns such that either $\bar{c}$ or $J$ (but not both) remains nearly fixed while the other varies as $1/\mathcal{L}$ or $\mathcal{L}$.

### Functional patterns

Having demonstrated and quantified how to induce composition patterns based on patterned in-plane strain/topography, we apply the same approach to designing composition patterns with targeted functional properties (electronic, photonic, etc.). For example, in-plane $J$ and/or $\bar{c}$ heterostructures and superlattices can be self-assembled by annealing alloy monolayers on substrates with prescribed topographies and/or in-plane strain patterns. Spatial patterns in $J$ can be constructed that, for example, correspond to spatial variations in direct versus indirect band gap for uses in photonic applications^62^.

Functional spatial patterns in $\bar{c}$ could be realized, for example, by pressing a monolayer onto a substrate with patterned wells (e.g., by drawing a vacuum to pull the regions suspended over the wells into a conformal state). This approach has been used to locally engineer strain and thus the band gap in WSe${}_{2}$, resulting in arrays of photoluminescent quantum dots^70^. Here, we propose using such localized strains to drive high $T$ composition evolution and self-assembly of in-plane $X$–$Y$ composition gradients and $M{X}_{2}$/$M{Y}_{2}$ heterostructures such as those shown in Fig. 2a (P1-right). Since spatial variations in both composition ($\bar{c}$ and $J$) and residual in-plane strains produce spatially varying bandgaps (see Fig. 2d), this approach can be used to create novel quantum structures, electronic heterojunctions, conductive interconnects, etc. Additionally, applying the same approach to the WTeS system, where variations in composition may produce a semiconductor to metal phase transformation^64^, a pattern of metallic “wires” can be created in a semiconducting sheet. This post-growth, self-assembly-based patterning approach is simpler and more spatially controllable than typical direct synthesis methods in which each material component of the heterostructure or superlattice is sequentially grown.

### Composition $\to$ shape programming

Now we examine how patterned compositions can be used to generate 3D structures from 2D sheets. Equilibrium composition $\to$ shape mappings are obtained by minimizing ${\mathcal{F}}$ with respect to $w(x,y)$ for fixed ${c}_{+}(x,y)$ and ${c}_{-}(x,y)$.

### Simple shapes

**Fig. 4:**
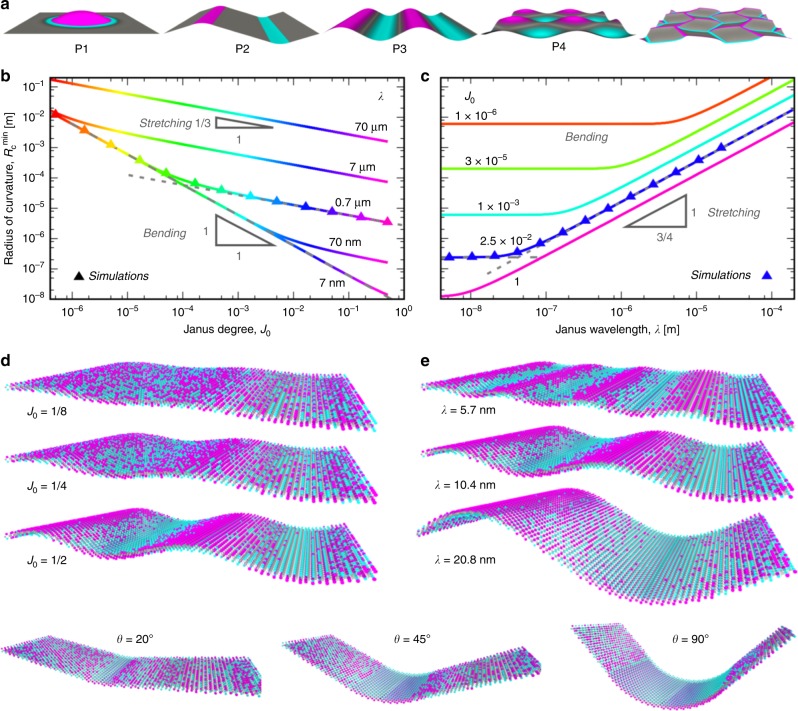
Composition $\to$ shape programming: 2D MoSeS monolayers that self-assemble into 3D shapes. **a** Topographies generated by the composition patterns displayed within each monolayer (also see Supplementary Movies 1–5). The composition patterns $J(x,y)$ for P1–P4 were generated using the expressions for bending-dominant composition $\to$ shape programming given in Table 1. Janus pattern color maps are scaled by their respective maxima ${J}_{0}$. **b**, **c** The minimum radius of curvature ${R}_{c}^{\min }$ for the sinusoidal profile P3 in **a** as a function of **b** Janus degree ${J}_{0}$ and **c** Janus wavelength for several values of $\lambda$ and ${J}_{0}$, respectively. The solid lines, dashed lines, and symbols are the full analytic result (see Methods), the asymptotic expansions in Table 1, and simulation results, respectively. Line colors correspond to Janus degree in both (**b**) and (**c**). **d**, **e** Atomic representations of the equilibrium shapes and compositions for MoSeS ($\bar{c}=1/2$) with 1D sinusoidal Janus patterns (P3) for several choices of **d** Janus degree (${J}_{0}$ at $\lambda =10.4$ nm) and **e** wavelength ($\lambda$ at ${J}_{0}=1/2$), and **f** with 1D ${J}_{0}=1/2$ Janus strips (P2) of width $L=1.9$, 5.8, and 11.6 nm

Analytical predictions of 3D shapes generated by different Janus composition patterns are reported in Table 1. Asymptotic results with respect to ${J}_{0}$ for the bending- and stretching-dominant regimes are provided; the latter are most relevant when the edges of the monolayer are clamped. The full expressions for $w(x,y)$ (rather than the asymptotic results) and the Janus degree at which the transition between bending and stretching-dominance occurs, ${J}_{{\rm{c}}}$, are provided in Methods and Supplementary Notes 3 and 4. Simulation results verifying the accuracy of these predictions and the fidelity of the resulting shapes are shown in Fig. 4a–c (also see Supplementary Movies 1–5). The minimum radius of curvature ${R}_{{\rm{c}}}^{\min }$ (${=}1/{w}_{xx}^{\max }=1/A{k}^{2}$) for the sinusoidal pattern P3 are shown in Figs. 4b, c. The results demonstrate that small-radius of curvature and/or large-amplitude features are most readily programmed when bending ($A \sim {J}_{0}$), rather than stretching ($A \sim {J}_{0}^{1/3}$), dominates.

### Scale dependence of shapes

As in shape $\to$ composition programming, the spatial dimensions of programmed topographies can be varied from the nano- to macro-scale, but features associated with $\bar{c}$ patterns and $J$ patterns scale differently with size. For a topography scaled equally in each dimension by a factor $\mathcal{L}$, $({L}_{x},{L}_{y},w)\to ({L}_{x},{L}_{y},w)\mathcal{L}$, the required bending eigenstrains (the product of the bending curvature and the length scale) are directly proportional to $\mathcal{L}$; ${w}_{ij}^{* }{\mathcal{L}} \approx 2J{\mathcal{L}}\check{\epsilon }{{\delta }_{ij}}/h$. A decrease in the scale of the pattern $\mathcal{L}$ thus requires an increase in $J$ and therefore energy density by the same proportion to maintain a given bending eigenstrain (i.e., shape). On the other hand, the stretching eigenstrain, ${\epsilon }_{ij}^{* }\approx \ {\delta }_{ij}\check{\epsilon }\delta \bar{c}$, has no such scale dependence; the size of a composition pattern containing only ${\epsilon }_{ij}^{* }$ can be varied with no change in energy density. Decreasing the scale $\mathcal{L}$ of a topographical pattern containing both bending and stretching eigenstrains requires no change in the $\bar{c}$ pattern but an increase in the Janus degree by a factor of $1/\mathcal{L}$.

### Programmed folds

Consider the case of 1D folds or bends; i.e., origami. The angle $\theta$ of a fold (i.e., the jump in the surface normal across the fold) programmed along ${\bf{y}}$ is set by the bending eigenstrain profile ${w}_{xx}^{* }$

12 $$\theta ={\int }_{-L/2}^{L/2}{w}_{xx}^{* }dx,$$

where $L$ is the width of the eigenbending profile. The fold angle produced by a strip of uniform Janus degree ${J}_{0}$ is $\theta ={w}_{xx}^{* }L=2{J}_{0}\check{\epsilon }L/h$ (see Fig. 4f). Other $J$ profiles can similarly produce folds; e.g., a Gaussian profile ${w}_{xx}^{* }=2{J}_{0}\check{\epsilon }{e}^{-{x}^{2}/2{\sigma }^{2}}/h$ can be used to create a fold of $\theta =\sqrt{8\pi }{J}_{0}\sigma \check{\epsilon }/h$. If the fold width ($L$ or $\sigma$ in the examples) is fixed in a particular material, design, or composition pattern, the required value of ${J}_{0}$ is uniquely determined.

The minimum fold width is physically constrained by the smallest $L$ that can be patterned, and the maximum fold angle for a given $L$ is constrained by the material-specific bound $| {J}_{0}| \le 1/2$. The smallest conceivable fold width is $L\ \approx \ a$, and the sharpest folds that can be realized at this scale ($| {J}_{0}| =1/2$) are $\theta \ \approx \ 2.{5}^{\circ }$, $3.{9}^{\circ }$, and $6.{7}^{\circ }$ for $M$SeS, $M$TeSe, and $M$TeS, respectively. On the other hand, the smallest fold widths capable of producing a $4{5}^{\circ }$ fold are $L\ \approx \ 6$, 4, and 2 nm for the same TMDs. We take these as proxies for the smallest feature sizes achievable with this approach, which implies that 3D objects as small as tens of nanometers are realizable.

### Complex shapes

As with shape $\to$ composition programming, in composition $\to$ shape programming, analytical predictions for simple shapes can be combined to create more complex shapes or numerical simulations can be employed to equilibrate the shape of a monolayer with fixed ${c}\!_{\pm }$. An example of the numerical approach is presented in the Discussion section.

### Uniformization theorem

In shape programming of sheets, the stretching and bending eigenstrains are general rank two tensors. However, in the TMDs considered here, the stretching and bending eigenstrain tensors are isotropic (i.e., changes in composition yield a distribution of point sources of dilatation and no shear). While this would seem to impose a restriction on the possible shapes that can be programmed, the Uniformization Theorem^71^ (an exact, geometric result that implies that any surface can be coordinatized with an isotropic metric) implies that there is no such restriction (in the limit that bending stiffness tends to zero). For this result to hold exactly, the system must admit arbitrarily large stretching eigenstrain (e.g., approaching infinity or $-1$ at discrete points). There is thus a restriction emerging from the maximum programmable stretching eigenstrain. 1D shapes such as P2 and P3, for example, cannot be isotropically coordinatized without extremely large stretching eigenstrains and in MoSeS must therefore contain unwanted misfit strain and shape distortion. The consequences of finite bending stiffness for achieving an exact target shape is higher order and can be at least partially resolved with programmed bending eigenstrain. This problem has been extensively studied in the computer graphics literature where algorithms have been developed to efficiently compute the required stretching eigenstrain field for a particular target shape^72^.

### Functional shapes

Building on the fundamental features of composition $\to$ shape programming in TMD monolayers outlined here, designs for shapes with targeted electronic, photonic, mechanical, and chemical properties can be created. These may include compliant corrugated designs for use in flexible electronics^46,49,73^, self-enclosing designs for storage and delivery of pharmaceuticals, soft robotics^44^, high surface area corrugated/crumpled monolayers for use in optical metasurfaces, light harvesting, and catalysis^24,74^, monolayers with geometric features designed for hydrophobic, hydrophilic, or omniphobic properties^74^, nanoplasmonic devices and sensors^47,55^, photodetectors^48^, and templates for selective self-assembly of molecules and nanoclusters^75^.

**Fig. 5:**
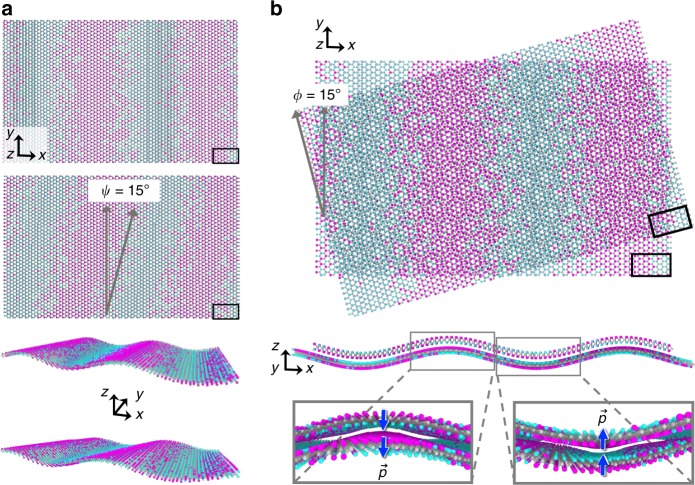
Programming the twist between TMD alloy monolayers with Janus patterns. **a** Atomic representations of two monolayers compositionally patterned along different crystallographic directions separated by an angle $\psi$. **b** When stacked, the twist angle $\phi$ is stabilized at $\psi$ by the preference to align the programmed out-of-plane deformations and electric dipole patterns of the two monolayers

Patterned and shaped monolayers may also be used to program the relative twist between stacked monolayers. When stacked, the crystallographic preference is to align the two monolayers with relative twist angle $\phi =0$. However, consider the stacking of two MoSeS monolayers with sinusoidal Janus patterns generated along different crystallographic directions an angle $\psi$ apart (see Fig. 5a). In this case, the Janus patterns introduce sinusoidal corrugations whose bending energy is minimized when the monolayers twist to $\phi =\psi$ and the corrugations align. Additionally, the electric dipoles created by the Janus stripes give rise to an electrostatic interaction that is also minimized when like dipole moments align between monolayers, again, at $\phi =\psi$. The relative twist angle can therefore be programmed to $\phi =\psi$ (see Fig. 5b) when the latter two effects outweigh the crystallographic preference for $\phi =0$.

Estimates of the energies involved (see Methods) suggest that twisted configurations may, indeed, be stable, particularly in $M$TeS. Janus stabilized twist would enable controlled study of the many interesting phenomena that emerge when 2D materials are stacked with particular twist angles, including unconventionally superconducting magic-angle superlattices^76^ and chiral stacks with plasmonic and other applications.

### Dynamic actuation with electric fields

**Fig. 6:**
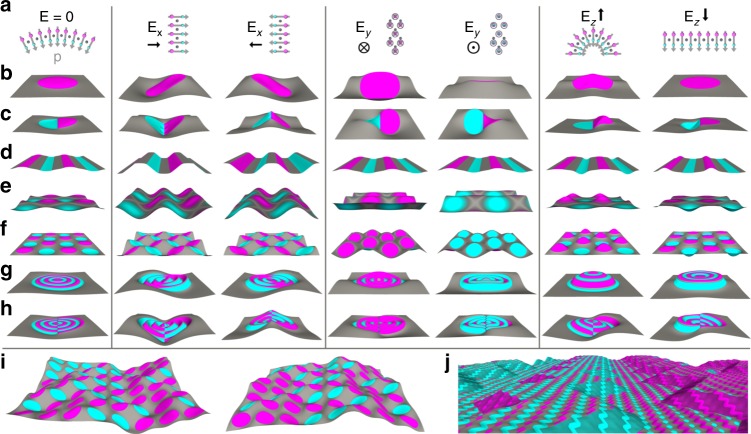
Using applied electric fields ${{\bf{E}}}_{{\rm{A}}}$ to actuate TMD alloy monolayers with composition-programmed shapes. **a** Schematics of how Janus regions in monolayers with clamped edges reorient, bend, or flatten according to their electric polarity relative to that of the field. Gray, magenta, and cyan spheres represent Mo, S, and Se atoms, respectively. Gray (black) arrows indicate atomic dipole moment (applied electric field) direction. The first column shows equilibrium shapes of seven Janus-patterned free standing sheets in the absence of an applied electric field (${{\bf{E}}}_{{\rm{A}}}=0$). **b**–**h** Simulated shapes of Janus-patterned MoSeS monolayers under uniform fields with directions shown in (**a**). **i**$\pm {{\bf{E}}}_{x}$-induced crumpling of a monolayer containing circular Janus domains arranged on a square lattice with randomly assigned polarity. **j** ${{\bf{E}}}_{x}$-induced two-scale buckling/folding of a patterned monolayer containing zigzag Janus domains on two length scales. Also see Supplementary Movies 6–26

Thin elastic sheets whose internal strains, and thus shape, can be altered using external stimuli (smart materials) are broadly useful in emerging technologies such as MEM/NEM actuation and soft robotics^77,78^. TMD alloy monolayers with nonzero Janus degree may be dynamically and reversibly reconfigured using external electric fields. Since Janus regions within a monolayer are electrically dipolar^60^, an applied electric field will exert local torques (${\boldsymbol{\tau }}={\bf{p}}\times {{\bf{E}}}_{{\rm{A}}}$) on Janus regions not aligned with the field. This produces local sheet reorientation, flattening, or bending, depending on the local electric polarity relative to that of the field (Fig. 6a).

### Actuation of patterned Janus monolayers

Janus patterns can be designed to exploit these effects and induce systematic, reversible, and dramatic shape changes with controlled application of electric fields. Examples of actuated shape change are shown in Fig. 6b–j. Embedded uniform Janus domains (Fig. 6b) follow in-plane fields ${{\bf{E}}}_{x}$ and ${{\bf{E}}}_{y}$ in a “sunflower” fashion and either pucker or flatten in response to ${{\bf{E}}}_{z}$ (Supplementary Movies 6 and 7). The realized shapes reflect a balance between the forces exerted by the electric field and induced elastic forces that tend to oppose the deformation (particularly in periodic monolayers). Embedded Janus domains with an axis of mirror symmetry, such as the elastically dipolar Janus-Janus circles shown in Fig. 6c, fold under ${{\bf{E}}}_{x}$ and distort into saddle-like shapes under ${{\bf{E}}}_{y}$ (Supplementary Movies 8–10). This folding effect can be implemented into mechanically anisotropic lamellar patterns that fold under one in-plane field direction (Fig. 6d, Supplementary Movie 11) or anisotropic 2D patterns that can fold in more than one direction but are geometrically frustrated from folding in certain directions (Fig. 6e, f, Supplementary Movies 12–18). Other examples include radial patterns of different symmetries (Fig. 6g, h, Supplementary Movies 19–24), lattices with randomly selected Janus polarity leading to quasi-random crumpling (Fig. 6i, Supplementary Movies 25 and 26), and actuatable multiscale or hierarchical patterns (Fig. 6j, Supplementary Movie 27). More complicated actuation effects can be achieved with nonuniform electric fields.

### Applications with actuation

Designs based on these dynamically configurable and reversible smart materials have potential uses in mechanical actuation of MEMS/NEMS and in dynamically controllable and tunable implementations of the applications noted in the previous section for designed static shapes. Properties of the materials and devices described therein can be rapidly and reversibly enabled, disabled, and tuned.

Potential applications based on the structures shown in Fig. 6 include unidirectional or multidirectional electromechanical actuators and force sensors (Fig. 6d, e, i), surfaces with dynamically variable multiscale features for tunable wettability (Fig. 6j), and dynamically reconfigurable surfaces for microfluidics and self-assembly templates (Fig. 6d–h, j)^79^. Additional uses include generating heterogeneous in-plane strains with $E$-fields (see, e.g., Fig. 6c) to dynamically and locally tune electronic structure, introducing Janus patterns and $E$-fields as degrees of freedom in design optimization for targeted mechanical and other properties, and employing $E$-field application protocols to guide sequential self-assembly of elaborate 3D shapes.

The forces generated during actuation can also be translated into propulsion mechanisms for nano/micro devices. For example, the flapping mode of Janus–Janus domains (Fig. 6c) could be incorporated into driven swimmers by adhering each half-circle onto a stiffer backing material. The force exerted by a perpendicular electric field on a Janus domain with one edge of width ${\ell}$ pinned in place is roughly $F \approx | {\bf{p}}| | {\bf{E}}|\ell$. A 1 MV m${}^{-1}$ electric field will therefore generate $\sim$10 pN of force over a $\ell =1$  µm domain, comparable to the locomotive forces generated by bacteria on similar length scales^80^. $F$ can be increased by stacking several monolayers.

## Discussion

We have demonstrated that shape and composition programmable TMD alloy monolayers are capable of self-assembling a wide variety of patterns and shapes with nano- to macro-scale features and that these shapes can be reversibly actuated with applied electric fields.

**Fig. 7:**
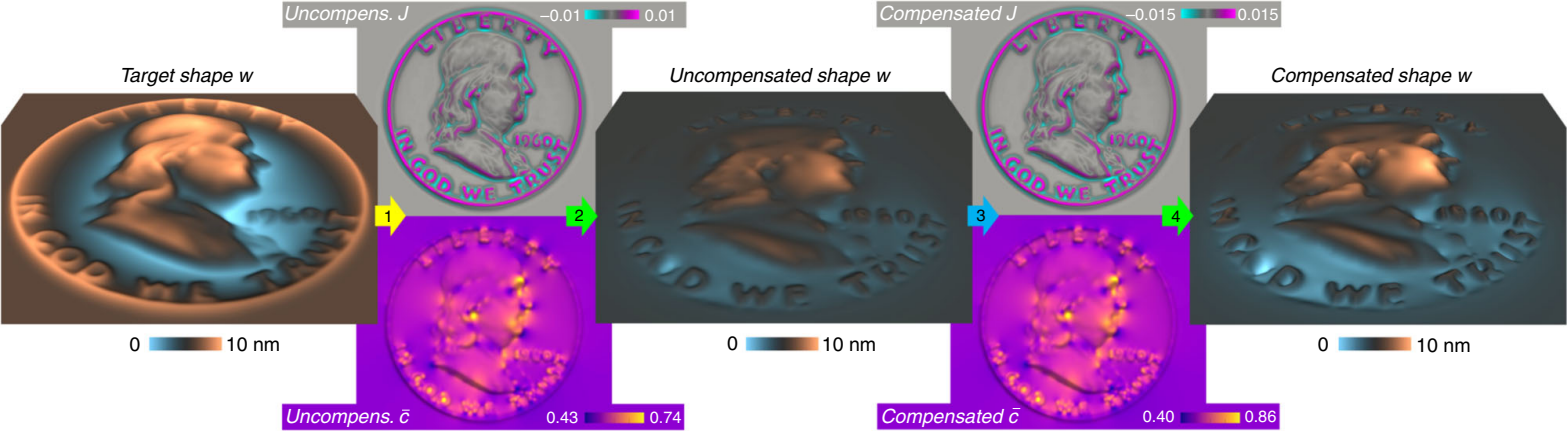
Benjamin MoSeS Franklin: a complex shape produced by numerical designed composition $\to$ shape programming. The target shape (left—a 1.4 µm version of the obverse of the Franklin half dollar) is used as a substrate template (1) upon which an initially homogeneous MoSeS monolayer is annealed at 1023 K (shape $\to$ composition programming, yellow arrow). (2) The resulting uncompensated composition state (“Uncompens.” $J$ and $\bar{c}$), upon release from the template yields an uncompensated shape with features very similar to the target but with smaller shape amplitude (composition $\to$ shape programming, green arrow). (3) The composition patterns obtained from (1) are multiplied by the ratio of target (10 nm) to uncompensated (6.7 nm) shape amplitude (compensation, blue arrow). (4) A monolayer assigned the compensated composition state, upon shape equilibration yields a shape with features and amplitude very similar to the target (composition $\to$ shape programming, green arrow)

The forward shape $\to$ composition and composition $\to$ shape programming processes examined above can be reposed as inverse or design problems; given a target shape or composition, determine the input composition or shape required to generate it. It is straightforward to invert our analytic solutions to the forward problems (see Table 1) to obtain solutions to the inverse problems for simple shapes/patterns. Our numerical solution approaches for complex shapes/patterns can also be adapted to these tasks by additionally optimizing shape amplitude with respect to target composition patterns (designed shape $\to$ composition) or composition magnitude with respect to target shape/topography (designed composition $\to$ shape). This optimization is accomplished in both cases by combining the two types of forward programming processes, as described in Methods and shown in Fig. 7 for a complex shape (the obverse of the US Franklin half dollar).

The relative bending and stretching strains (or $J$ and $\bar{c}$ pattern magnitudes) associated with a target shape can significantly affect programmed shape accuracy. The accuracy with which complex shapes such as the Benjamin MoSeS Franklin example shown in Fig. 7 can be programmed increases as the amplitude of the target shape decreases. Equilibrium composition patterns become $J$-dominated at small amplitude. However, the topographical amplification factor simultaneously approaches its maximum value (bending-dominant $1+{\Delta }_{w}\ \approx \ 7.6$ at $T=1023$ K), such that the realized free-standing shapes become increasingly flattened relative to their templates. On the other hand, the composition patterns that produce large amplitude complex shapes tend to be $\bar{c}$-dominated, which results in less flattening upon removal from the template substrate but less overall shape accuracy.

Our numerical procedures for designed programming can be combined to enable physical realization in the laboratory. For example, once a composition pattern that will program a target shape has been determined by our designed composition $\to$ shape programming procedure, this composition pattern must be physically realized. A substrate template topography that will produce the desired pattern can be computed using the designed shape $\to$ composition programming procedure described above.

Recent experimental results demonstrate that the fundamental mechanisms behind our approach are operable and observable. A link between composition and shape has been established, e.g., in defect-free WS${}_{2}$/WSe${}_{2}$ superlattice crystals^26^ and coherent MoS${}_{2}$/MoTe${}_{2}$ monolayer heterostructures^27^, where compositional misfit strain created by S/Se and S/Te gradients induces periodic out-of-plane ripple patterns. The production of stable Janus MoSeS monolayers^61–63^ further indicates that Janus degree can be spatially controlled. The viability of the topographic annealing approach that we propose for this purpose (shape $\to$ composition programming) is supported by experimental results which demonstrate that transition metal diffusivities are sufficiently large (at TMD growth temperatures) to redistribute transition metal composition profiles under relatively small driving forces^81^. Since chalcogen diffusivities are generally larger than metal diffusivities, this suggests that significant chalcogen redistribution will also be achievable.

Nanopatterned TMD growth substrate topographies have been demonstrated using ion-beam-projection lithography^23^. TMD synthesis on these topographically anisotropic substrates resulted in conformal, isomorphic nanosheet growth. This demonstrates that TMD properties can be tailored via topography-based strain engineering and potentially provides a means to spatially control Janus degree in TMD alloys.

The model presented in this work describes single crystal monolayers without grain boundaries or other topological defects. This limits its validity to single grains, which can currently be grown over $\sim$10 µm in size. The presence of toplogical defects will couple to and alter strains and compositions. For example, during monolayer growth on non-flat surfaces, shape strain can be accommodated by building topological defects into the lattice. This suppresses the mechanism of shape $\to$ composition programming, indicating that high $T$ annealing rather than synthesis on non-flat surfaces should be the more effective approach for topographical shape $\to$ composition programming. If monolayers containing grain boundaries or domain walls are used, we expect our description to remain valid when strains are sufficiently small and/or grain sizes are sufficiently large relative to topographical or compositional feature sizes. When these conditions are not met, local compositional heterogeneities associated with grain boundaries may become more significant than those created by topographic patterns, and strain-driven defect migration during transfer and annealing may plastically relieve some topographic strain and partially suppress composition patterning. The effects of topological defects can however be built into our model, e.g., as additional contributions to the eigenstrain fields ${\epsilon }_{ij}^{* }$ and ${w}_{ij}^{* }$. Effects associated with finite substrate/monolayer interaction strengths (e.g., slipping and delamination) and free sheet edges can also be treated with appropriate modifications.

The crux of the shape/composition programming paradigm is the determination of the fundamental relations between composition and deformation. We derived these relations for our proposed atomically thin smart shape/composition programming platform and have also presented a physical model with which the relations can be numerically computed for complicated shapes and patterns. Our modeling approach is sufficiently general for application to other TMD alloy systems, other 2D materials, and other shape/composition programmable materials. While our focus here has largely been on shape and composition programming, the ultimate goal is physical property programming - optimization of patterns and shapes with respect to target material properties. We have provided examples of spatial bandgap modulation and have suggested several other possibilities involving designed mechanical, catalytic, chemical, thermal, and optical properties. Combination of the utility of smart shape programmables with the diversity of intrinsic TMD properties and designed composition patterns, all tunable across length scales, suggest a broad set of potential applications for this 2D material platform.

## Methods

### Plate theory formalism

Here we outline the derivation of the modified Föppl-von Kármán equations used in this work, Eq. (2). These equations include the effects of general eigenstretching and eigenbending fields.

We describe material deformation mathematically as two embeddings of the 2D (with small thickness) material in 3D space (Table 2). One embedding represents the undeformed geometry and gives the Lagrangian, or material, coordinates (notated in upper case). The other represents the deformed geometry and gives the Eulerian, or spatial, coordinates (notated in lower case). The indices $a,b,c,\ldots$ range over $\{1,2,3\}$, describing the full three dimensional coordinates, while the indices $i,j,k,\ldots$ range over $\{1,2\}$, describing the approximation of the plate as two dimensional. We use the Lagrangian convention, where deformation is said to take a point ${X}_{a}$ to ${x}_{a}({X}_{a})$.

**Table 2:** Geometric embeddings

	2D Coordinates	3D Coordinates
Lagrangian	${X}_{i}=({X}_{1},{X}_{2})$	${X}_{a}=({X}_{1},{X}_{2},{X}_{3})$
Eulerian		${x}_{a}=({x}_{1},{x}_{2},{x}_{3})$

To describe stretching, we consider first the in-plane stretching deformation in the $({X}_{1},{X}_{2})$ coordinate basis. The displacement vector in 3D Lagrangian coordinates is defined ${u}_{a}({X}_{i})={x}_{a}({X}_{i})-({X}_{i},0)$. The full Lagrangian stretching deformation tensor is

13 $${\epsilon }_{ij}({X}_{i})=\frac{1}{2}({u}_{i,j}+{u}_{j,i}+{u}_{a,i}{u}_{a,j}).$$

For bending, we consider the small but finite thickness of the plate to find the moments that give the bending deformation. We use the Kirchoff-Love hypothesis and other approximations to give that strains ${\epsilon }_{3a}$ are small and that the gradient ${x}_{a,3}({X}_{i})$ is parallel to the normal vector of the plate, ${n}_{a}({X}_{i})$. Thus, the full deformation is ${x}_{a}({X}_{a})={x}_{a}({X}_{i})+{X}_{3}{n}_{i}({X}_{i})$. The full in-plane stretching deformation is ${\epsilon }_{ij}({X}_{a})={\epsilon }_{ij}({X}_{i})-{X}_{3}{w}_{ij}$. The term ${w}_{ij}={x}_{a,ij}{n}_{a}$ is the bending deformation tensor, commonly known as the curvature tensor or the second fundamental form of the surface.

The approximation of the Föppl-von Kármán equations is that the plate undergoes small deformations and small rotations out of plane. Thus, we ignore the higher order terms $O({u}_{i,j}^{2})$ and the normal vector ${n}_{a}$ is nearly entirely in the out-of-pane direction ${X}_{a,3}$. For convenience, we notate the out-of-plane component ${u}_{3}$ as $w({X}_{i})={u}_{3}({X}_{i})={x}_{3}({X}_{i})$. With this, the stretching and bending deformation tensors become

14 $${\epsilon }_{ij}({X}_{i})=\frac{1}{2}({u}_{i,j}+{u}_{j,i}+{w}_{,i}{w}_{,j}+O({u}_{i,j}^{2}))\approx \frac{1}{2}({u}_{i,j}+{u}_{j,i}+{w}_{,i}{w}_{,j})$$

and

15 $${w}_{ij}={x}_{a,ij}{n}_{a}\approx {x}_{3,ij}={w}_{,ij}.$$

The following definitions are employed for strain. Strain is the deformation from the state that has strain zero. Eigenstrain ${u}_{ij}^{* }/{w}_{ij}^{* }$ is the deformation from the reference state to the state with strain zero. Deformation ${u}_{ij}/{w}_{ij}$ is the deformation from the reference state.

Thus, the strain tensor at a point ${X}_{i}$ is

16 $${\epsilon }_{ij}({X}_{i})-{\epsilon }_{ij}^{* }({X}_{i})-{X}_{3}({w}_{ij}({X}_{i})-{w}_{ij}^{* }({X}_{i})).$$

The usual elastic energy functional ${\mathcal{F}}=\int \int \frac{1}{2}{\lambda }_{ijkl}{\epsilon }_{ij}{\epsilon }_{kl}$ is replaced by substituting ${\epsilon }_{ij}$ for our full expression for the strain with eigenstrain to obtain

17 $${\mathcal{F}}=\int \int \frac{1}{2}{\tilde{\lambda }}_{ijkl}\left[h{(u-{u}^{* })}_{ij}{(u-{u}^{* })}_{kl}+\frac{{h}^{3}}{12}{(w-{w}^{* })}_{ij}{(w-{w}^{* })}_{kl}\right].$$

### Electric dipoles

We examine the importance of the dipole-dipole interaction energy in MoSeS monolayers by examining its effect on pattern P2, a 1D quadratic bend ($w=B{x}^{2}/2$) created by a thin homogeneous Janus strip, in this case within a monolayer with free edges. As can clearly be seen in Supplementary Fig. 1, the effect of dipole–dipole interactions is not found to qualitatively alter the results obtained in the absence of these electrostatic effects. We therefore neglect dipole–dipole interactions in this work, which significantly simplifies the analysis and simulations.

Employing this approximation, the thermodynamic (dipolar) potential that dictates the monolayer’s topography in electrostatic equilibrium under an applied electric field ${{\bf{E}}}_{{\rm{A}}}$ is

18 $$\frac{\delta {{\mathcal{F}}}_{{\rm{electric}}}}{\delta w}=	-\frac{2{p}_{0}}{{Z}^{5/2}}{\mathop{\sum}\limits _{i=x,y}}\; {\mathop{\sum}\limits _{j=x,y}^{j\ne i}} \left[{E}_{i}\left(Z\left[{J}_{,i}({Z}_{jj}+1)-{J}_{,j}{Z}_{ij}\right]-J\left[2{w}_{,j}{w}_{,ij}(1-2{Z}_{ii}+{Z}_{jj})\right.\right.\right.\\ 	\left.\left.\left. +\, {w}_{,i}\left(3{w}_{,ii}({Z}_{jj}+1)+{w}_{,jj}(1-2{Z}_{jj}+{Z}_{ii})\right)\right]\right)\right.\\ 	+ \left.{E}_{z}\left(Z{J}_{,i}{w}_{,i}-J\left[{w}_{,ii}(2{Z}_{ii}+{Z}_{jj}-1)+3{w}_{,ij}{Z}_{ij}\right]\right)\right]$$

where $$Z={({w}_{,x})}^{2}+{({w}_{,y})}^{2}+1$$, $${Z}_{ii}={({w}_{,i})}^{2}$$, $${Z}_{jj}={({w}_{,j})}^{2}$$, and $${Z}_{ij}={w}_{,i}{w}_{,j}$$.

### DFT calculations

Here, we detail the DFT calculations performed to determine $${f}\!_{{\rm{mix}}}({c}\!_{\pm })$$ and $$\Lambda$$ for MoSeS. All DFT calculations were performed with the Vienna Ab Initio Simulation Package (VASP)^82,83^ using a plane-wave basis set and the projector augmented wave method^84,85^. The Perdew-Burke-Ernzerhof generalized gradient approximation was employed to treat exchange-correlation effects^86^. The plane wave kinetic energy cutoff was set to 420 eV, and a $$12\times 12\times 1$$$$\Gamma$$-centered k-point grid was employed for Brillouin zone integration. All atomic structures were fully relaxed until the total force on each atom was $$<0.01$$ eV Å$${}^{-1}$$. A vacuum layer of minimum thickness 20 Å perpendicular to the monolayer was employed to minimize interactions between the monolayer and its periodic images.

The total energies of $M{X}_{2}$ and $M{Y}_{2}$ (${U}_{X}$ and ${U}_{Y}$) appearing in the enthalpy of mixing were directly computed (Supplementary Table 1). Their derivatives at $\bar{c}=0$ and 1 were evaluated from the change in total energy density upon substituting one Se (S) atom into large supercells containing 150 atoms ($5\times 5$ conventional unit cells). Results of these calculations are shown in Supplementary Fig. 2a. We obtained $dU/d\bar{c}{| }_{\bar{c}=0}=({U}_{\bar{c}=0.01}-{U}_{\bar{c}=0})/0.01=1.666$ eV per Mo and $dU/d\bar{c}{| }_{\bar{c}=1}=({U}_{\bar{c}=1}-{U}_{\bar{c}=0.99})/0.01=1.951$ eV per Mo. Using these values, we determined the regular solution parameter; $\chi =(dU/d\bar{c}{| }_{\bar{c}=0}-dU/d\bar{c}{| }_{\bar{c}=1})/2=-0.1425$ eV per Mo.

The constant that describes the compositional interactions between chalcogen layers, $\Lambda$, was determined by considering monolayers with only S atoms in one chalcogen layer and random mixtures of S and Se atoms at different average compositions in the other chalcogen layer. $\Lambda$ was quantified from the difference in total energy between such asymmetric monolayers ${U}^{a}$ and symmetric monolayers with equal compositions in both chalcogen layers ${U}^{s}$. We fit these data to a quadratic form ${U}^{a}-{U}^{s}\ \approx \ ({U}_{2}^{a}-{U}_{2}^{s}){({\bar{c}}_{+}-{\bar{c}}_{-})}^{2}$ (see Supplementary Fig. 2b). Comparison with Eq. (5) shows that $\Lambda =2({U}_{2}^{a}-{U}_{2}^{s})\ \approx \ 0.13$ eV per Mo-atom.

### Mechanical and chemical relaxation

Out-of-plane displacements are evolved toward equilibrium using a physically-motivated damped wave equation that approximates flexural acoustic phonons

19 $${w}_{,tt}+\left({\beta }_{0}-{\beta }_{1}{\nabla }^{2}\right){w}_{,t}=-{\alpha }_{w}^{2}\frac{\delta {\mathcal{F}}}{\delta w}.$$

${\beta }_{0}$ and ${\beta }_{1}$ are damping parameters that operate uniformly and preferentially on large wavenumber oscillations, respectively, and ${\alpha }_{w}$ is the propagation speed of bending waves.

For conditions in which chalcogen composition is not conserved, ${c}_{+}$ and ${c}_{-}$ are dissipatively relaxed to equilibrium

20 $${c}\!_{\pm ,t}=-{M}\!_{\pm }\left(\frac{\delta {\mathcal{F}}}{\delta {c}\!_{\pm }}-{\lambda }_{0}\right).$$

Here, ${M}\!_{\pm }$ is the diffusional mobility of chalcogens and ${\lambda }_{0}$ is the environmental chemical potential. This choice assumes substitutional diffusion between species $X$ and $Y$ (${M}_{X}={M}_{Y}={M}\!_{\pm }$).

For conditions in which chalcogen composition is conserved but only equilibrium states are of interest (physical kinetics are not necessary), a Lagrange multiplier is added to enforce conservation without the need for slower diffusive kinetics

21 $${c}\!_{\pm ,t}=-{M}\!_{\pm }\left(\frac{\delta {\mathcal{F}}}{\delta {c}\!_{\pm }}-{\lambda }_{\pm }\right).$$

Here, $${\lambda }_{\pm }={A}^{-1}{\int }_{A}(\delta F/\delta {c}\!_{\pm })d$$$${\bf{r}}$$ is the Lagrange multiplier that enforces global conservation of species in each $$XY$$-layer.

Conserved composition and physical kinetics are treated with generalized diffusion equations

22 $${c}\!_{\pm ,t}={M}\!_{\pm }\nabla \cdot \left({c}\!_{\pm }\nabla \frac{\delta {\mathcal{F}}}{\delta {c}\!_{\pm }}\right)\approx {M}\!_{\pm }{\nabla }^{2}\frac{\delta {\mathcal{F}}}{\delta {c}\!_{\pm }}.$$

### Composition-to-twist programming

An example of Janus-stabilized twist, $\phi$, between two MoSeS monolayers with sinusoidal Janus patterns programmed along crystallographic directions separated by $\psi =1{5}^{\circ }$ is shown in Fig. 5. The energy increase between $\phi =0$ (untwisted) and large $\phi$ (twisted) states has been calculated for MoS${}_{2}$ bilayers from first principles^87^ to be $\approx 0.02$ Jm${}^{-2}$. For Janus patterned bilayers with ${\psi}\, {\ne}\, {0}$, the dipole anti-alignment imposed by Janus-misaligned states (${\phi} \,{\ne} \,{\psi}$) leads to an electrostatic energy decrease in the twisted, Janus-aligned state ($\phi =\psi$) of $\approx {(2{p}_{0}{J}_{0})}^{2}/2\pi {\epsilon }_{0}{d}_{b}^{3}\ \approx \ 6\times 1{0}^{-4}$ Jm${}^{-2}$ for ${J}_{0}=1/2$ (${\epsilon }_{0}=8.85\times 1{0}^{-12}$ Fm${}^{-1}$ is the permittivity of air or vacuum and ${d}_{b}\,{\approx} \,0.63$ nm is the distance between stacked MoSeS monolayers). This energy decrease is not sufficient to stabilize large twist angles. However, our estimates indicate that the bending energy decrease between Janus-misaligned and twisted, Janus-aligned MoSeS states is $\gtrsim 0.023$ Jm${}^{-2}$ for monolayers much larger than the Janus wavelength, which may be sufficient to stabilize twisted configurations. Our calculations also indicate that this twist-stabilizing bending energy decrease is linearly proportional to both $\kappa$ and $\check{\epsilon }$. This suggests that Janus pattern-induced twist-stabilization will be most effective in $M$TeS, which has ${\sim}$10% larger $\kappa$ and ${\sim}$100% larger $\check{\epsilon }$ than $M$SeS.

The bending energy estimates above are obtained from Eq. (4) as the difference between the bending energy minimum of both monolayers in their respective bending-relaxed states (when $\phi =\psi$) and the bending energy maximum of one monolayer in its bending-relaxed state and the other forced to conform to the first (against its programming, when ${\phi}\, {\ne}\, {\psi}$). More detailed calculations are required to fully address the question of under exactly which conditions such twisted structures will be stable.

### Inverse/designed shape $\to$ composition programming

Designed shape $\to$ composition programming begins with (1) a forward composition $\to$ shape step; the target composition pattern is assigned to a flat monolayer and its shape $w(x,y)$ is equilibrated. Then (2) a forward shape $\to$ composition step is performed by fixing a compositionally homogeneous monolayer in the shape obtained in (1) and equilibrating composition. The magnitude of the resulting composition pattern will differ from that of the target by a factor of ${\sim} {(1+\Delta )}^{-1}$, where $\Delta$ is the chemo-elastic ratio of the shape obtained in (1). Multiplying the shape amplitude obtained in (1) by the value of $1+\Delta$ obtained in (2) gives the compensated substrate template amplitude. While only one iteration of this procedure is typically required to obtain high fidelity substrate amplitudes, the procedure can be iterated to the required degree of convergence.

### Inverse/designed composition $\to$ shape programming

Analogously, designed composition $\to$ shape programming begins with (1) a forward shape $\to$ composition step; a monolayer is fixed into the target shape and its composition is equilibrated. Then (2) a forward composition $\to$ shape step is performed by assigning the composition pattern obtained in (1) to a flat monolayer and equilibrating its shape. The amplitude of the resulting shape will differ from that of the target by a factor of $\sim {(1+\Delta )}^{-1}$, where $\Delta$ is the chemo-elastic ratio of the target shape. Multiplying the pattern magnitudes obtained in (1) by the value of $1+\Delta$ obtained in (2) gives (3) the compensated composition pattern magnitudes, which (4) upon shape equilibration produce an improved programmed shape. This procedure can be iterated until the required shape amplitude accuracy is achieved. Figure 7 shows a complex shape (the obverse of the US Franklin half dollar—i.e., Benjamin MoSeS Franklin) programmed into a $\bar{c}=1/2$ MoSeS monolayer using this approach.

## Supplementary information

Supplementary Information

Description of Additional Supplementary Files

Supplementary movie 1

Supplementary movie 2

Supplementary movie 3

Supplementary movie 4

Supplementary movie 5

Supplementary movie 6

Supplementary movie 7

Supplementary movie 8

Supplementary movie 9

Supplementary movie 10

Supplementary movie 11

Supplementary movie 12

Supplementary movie 13

Supplementary movie 14

Supplementary movie 15

Supplementary movie 16

Supplementary movie 17

Supplementary movie 18

Supplementary movie 19

Supplementary movie 20

Supplementary movie 21

Supplementary movie 22

Supplementary movie 23

Supplementary movie 24

Supplementary movie 25

Supplementary movie 26

Supplementary movie 27

## Data Availability

The data supporting the findings of this study are available within the paper and its Supplementary files, and are available from the corresponding author upon request.

## Code availability

The custom C/MPI code used in numerical simulations is available from the corresponding author upon reasonable request.
