# Supplementary material for: The MoSeS dynamic omnigami paradigm for smart shape and composition programmable 2D materials
Source: Nat Commun. 2019 Nov 15;10:5210. doi: 10.1038/s41467-019-12945-5 (PMC6858317; doi:10.1038/s41467-019-12945-5)
Supplement: Supplementary file 1 — Supplementary Information [file 41467_2019_12945_MOESM1_ESM.pdf]

Supplementary Information

The MoSeS Dynamic Omnigami paradigm for smart shape and composition programmable 2D materials

Berry et al.

Supplementary Figures

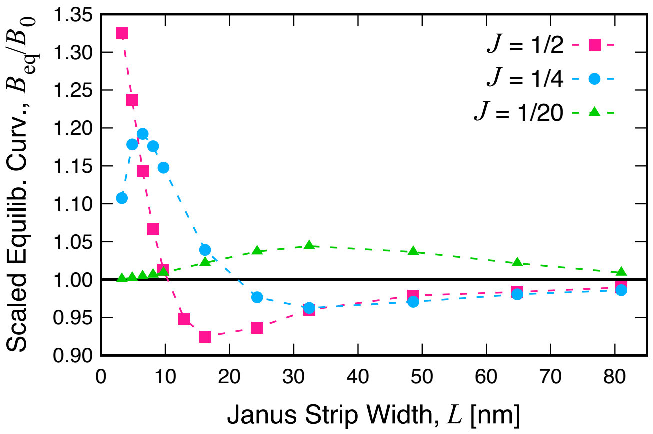

Supplementary Figure 1 | Effect of dipole-dipole interactions on the equilibrium shapes of MoSeS. The equilibrium curvature, B_{eq}, of a quadratic 1D bend (pattern P2) scaled by the equilibrium curvature in the absence of electrostatic effects, B_0, is plotted versus the Janus strip width L for three values of J.

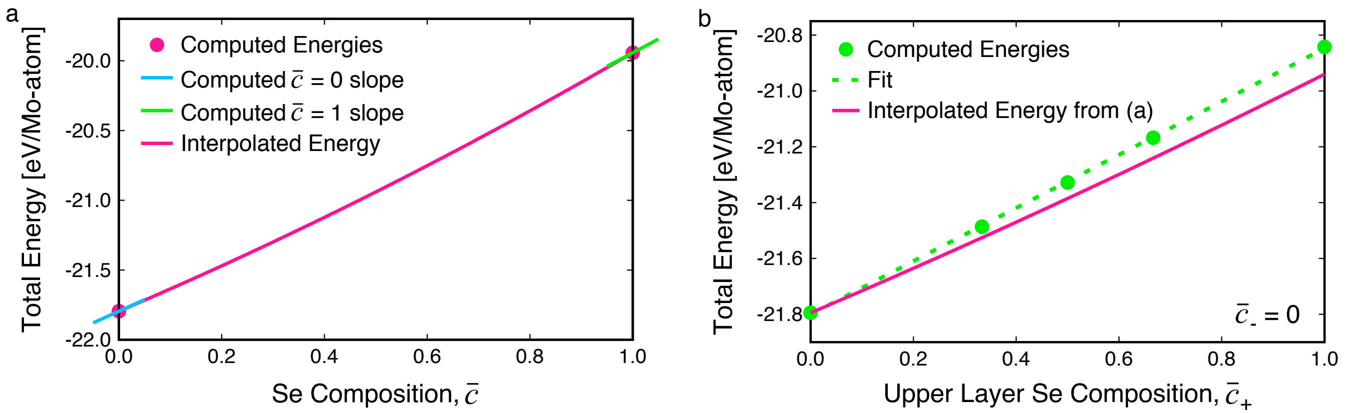

Supplementary Figure 2 | DFT parameterization of MoSeS monolayer alloy thermodynamics. a Fit function for $f_{\mathrm{mix}}(\bar{c})$ in a random solid solution alloy. The magenta line is the fit interpolated between the computed slopes at $\bar{c} = 0$ (short blue line) and 1 (short green line). **b** Total energy vs. $\bar{c}_+$ for partial Janus alloys with $\bar{c}_- = 0$ (right). The magenta line is the same as that in the left figure (random MoSeS alloy), and the dashed green line is the fit to the green data points (Janus MoSeS). The interplane compositional interaction constant Λ is determined from the difference between the two lines.

Supplementary Tables

Supplementary Table 1 | Descriptions of notation and model / material input parameters.

M	Transition metal species, Mo or W
X	Larger chalcogen species, Se or Te
Y	Smaller chalcogen species, S or Se
$\mathcal{F}$	Free energy
c_+	Composition field of X in upper chalcogen layer
c_-	Composition field of X in lower chalcogen layer
$\bar{c}_+$	Average composition of X in upper chalcogen layer
$\bar{c}_-$	Average composition of X in lower chalcogen layer
$\bar{c}$	Average composition of X in both chalcogen layers
$\bar{c}_0$	Average composition of the eigenstrain reference state (a stress-free undeformed monolayer)
$\delta\bar{c}$	Average local deviation from reference composition
J	Janus degree field: $(c_+ - c_-)/2$
f_{mix}	Energy of mixing within a given chalcogen layer
χ	Regular solution parameter [-0.1425 eV per Mo]
Λ	Constant characterizing the strength of compositional interaction between the two chalcogen layers [0.13 eV per Mo]
γ	Compositional interface energy constant [10^{-10} eV$^{1/2}$]
μ_0	Strength of external field conjugate to composition
U_Z	Total energy density of MZ_2 [$U_{\text{Se}} = -19.943$ eV per Mo , $U_{\text{S}} = -21.795$ eV per Mo]
U^s	Total energy density of a symmetric monolayer
U^a	Total energy density of an asymmetric monolayer
$\tilde{k}_{\text{B}}$	Boltzmann's constant per unit area
T	Temperature
a	Lattice parameter, zigzag direction [$a_X\bar{c} + (1-\bar{c})a_Y$, $a_X = 0.318$ nm, $a_Y = 0.332$ nm[1]]
b	Lattice parameter, armchair direction [$b_X\bar{c} + (1-\bar{c})b_Y$, $b_X = 0.551$ nm, $b_Y = 0.575$ nm[1]]
h	Monolayer geometric (physical) thickness [0.32 nm[2]]
$\tilde{h}$	Monolayer elastic (effective) thickness [$(12(1-\nu^2)\kappa/E)^{1/3} = 0.46$ nm]
λ_{ijkl}	Elastic stiffness tensor, $i, j, k, l \in \{x, y\}$
E	Young's modulus [180 GPa[3]]
ν	Poisson's ratio [0.25[3]]
$\tilde{\nu}$	$2(1+\nu)$
κ	Bending stiffness [10 eV[4]]
u_i	In-plane displacement fields, $i \in \{x, y\}$
w	Monolayer out-of-plane displacement field
ϵ_{ij}	Elastic stretching deformation tensor
$w_{,ij}$	Second partial derivatives of w, the curvature tensor
ϵ^*_{ij}	In-plane misfit strain or eigenstrain
w^*_{ij}	Local spontaneous curvature tensor
$\epsilon_{ij} - \epsilon^*_{ij}$	Elastic strain
$\check{\epsilon}$	Maximum eigenstrain: $(a_X - a_Y)/a_X$ [0.044[1]]
$\bar{\epsilon}_{ij}$	Macroscopic applied strain tensor
$\mathbf{E}_{\text{A}}$	Applied electric field
$\mathbf{p}$	Dipole moment per XY pair
p_0	Dipole moment density at $J = 1/2$ [0.052 eÅ per dipole[2]]
$\hat{\mathbf{n}}$	Local normal vector of the monolayer
E_{g}	Electronic bandgap
E^Z_{g}	Electronic bandgap of MZ_2 at zero strain
ΔE^Z_{ii}	Change in E^Z_{g} with axial strain ϵ_{ii}
J_0	Amplitude of Janus degree pattern

Supplementary Table 2 | Descriptions of computed material parameters.

Δ_w	Chemo-bending ratio, ratio of chemical to elastic bending energies that quantifies compositional suppression of inhomogeneous spatial Janus patterns
Δ_ϵ	Chemo-stretching ratio, ratio of chemical to elastic stretching energies that quantifies compositional suppression of inhomogeneous spatial alloy patterns
$1+\Delta_w$	Topographical amplification factor for shape $\rightarrow$ composition programming
$1+\Delta_\epsilon$	Stretching amplification factor for shape $\rightarrow$ composition programming
J_c	Crossover J_0 between bending- and stretching-dominant regimes in clamped-edge monolayers
α	Elastic prefactor for stretching-dominant shape $\rightarrow$ composition programming
β	Constant related to maximum in-plane eigenstrain
E_ϵ	Ratio of stretching to bending terms in stretching-dominated composition-programmed shapes
E_c	Characteristic constant for the crossover between bending- and stretching-dominated regimes in composition $\rightarrow$ shape programming
R_c	Equilibrium radius of curvature
θ	Equilibrium angle of a 1D bend produced by a uniform Janus strip (P2)

Supplementary Notes

Supplementary Note 1: Dipole-Dipole Interactions

Here, we assess the importance of the dipole-dipole interaction energy in MoSeS monolayers by examining its effect on pattern P2, a 1D quadratic bend ($w = Bx^2/2$) created by a thin homogeneous Janus strip, in this case within a monolayer with free edges. The equilibrium curvature of the bend, B_{eq}, is computed by numerically minimizing $\mathcal{F}_w + \mathcal{F}_{\mathrm{electric}}$ with respect to B in the presence of dipole-dipole interactions.

The results as a function of the strip width L are shown in Fig. 1 for three values of J. Dipole-dipole effects are only notable (induce $> 5\%$ deviation) in monolayers with very small features ($L \lesssim$ 10-20 nm) and large Janus degrees ($J \gtrsim 0.05$). Their effect is not found to qualitatively alter the results obtained in the absence of electrostatic effects. We therefore neglect dipole-dipole interactions in this work, which significantly simplifies the analysis and simulations.

Supplementary Note 2: Spatially Heterogeneous Bandgap Mappings

The bandgap maps shown in Fig. 2d are calculated assuming bandgap varies linearly with $\bar{c}$ [5], is unaffected by J [2], and that the variation with homogeneous strain is applicable to heterogeneous strain states;

$E_{\mathrm{g}} = \max\{(1-\bar{c})(E_{\mathrm{g}}^Y + \Delta E_{ii}^Y) + \bar{c}(E_{\mathrm{g}}^X + \Delta E_{ii}^X), 0\}$, where E_{g}^Z is the zero-strain bandgap of MZ_2 and ΔE_{ii}^Z is the variation of the MZ_2 bandgap with axial strain ϵ_{ii}. For MoSeS, first principles calculations[6] give $\Delta E_{ii}^{\mathrm{S}} \approx -9.4\epsilon_{ii}$ for $-0.01 \leq \epsilon_{ii} \leq 0.1$, $\approx 3.7\epsilon_{ii}$ for $-0.09 \leq \epsilon_{ii} < -0.01$, and $\Delta E_{ii}^{\mathrm{Se}} \approx -6.1\epsilon_{ii}$ for $0 \leq \epsilon_{ii} \leq 0.12$, $\approx 2.2\epsilon_{ii}$ for $-0.05 \leq \epsilon_{ii} < 0$. Strain is defined relative to the delimiting $M\mathrm{S}_2$ and $M\mathrm{Se}_2$ states. Shear strain effects are small and neglected here. These predictions are subject to uncertainty from the assumptions noted above and from accuracy limits of the referenced DFT calculations. The states in the 4th and 5th panels in Fig. 2d exhibit larger strain variations and thus larger E_{g} variations than the states in the other panels.

Supplementary Note 3: Full Composition $\rightarrow$ Shape Programming Mappings

The equilibrium composition-programmed shapes (geometric amplitudes) generated by various Janus patterns and

applicable across both elastic regimes are presented here. For a uniform Janus circle (P1), $J = J_0 H(r_0 - \sqrt{x^2+y^2})$,

$$\frac{r_0^2}{R} = \frac{6J_0\check{\epsilon}r_0^2}{h}\frac{\Phi\sqrt{\Upsilon}}{\left(\sqrt{\Upsilon}+\Phi\right)^2} \tag{1}$$

where

$$\Upsilon = \left(-K_J - \Phi^3 + \sqrt{K_J(K_J+2\Phi^3)}\right)^{2/3}, \tag{2}$$

$$\Phi = (1-\nu^2)(2h^2\kappa/r_0^4)^{1/3}, \tag{3}$$

and

$$K_J = 12[J_0\check{\epsilon}(1-\nu^2)]^2\tilde{h}E. \tag{4}$$

For a uniform Janus strip (P2), $J = J_0 \mathrm{rect}(x/L)$,

$$BL^2 = \frac{L^2}{6(1-\nu)h\sqrt{\tilde{h}E}}\frac{\Phi(\Upsilon-\Phi)}{\sqrt{\Upsilon}}, \tag{5}$$

where

$$\Upsilon = \left(\sqrt{K_J} + \sqrt{K_J+\Phi^3}\right)^{2/3}, \tag{6}$$

$$\Phi = 4(1-\nu)\left(6\kappa h^2/L^3 d\right)^{1/3}, \tag{7}$$

and

$$K_J = 4[9J_0\check{\epsilon}(1-\nu^2)]^2\tilde{h}E. \tag{8}$$

For an nD sinusoidal Janus pattern (P3, P4), $J = J_0\sum_{i=1}^{n}\sin(kx_i)/n$,

$$A = \sqrt{\frac{1+\nu}{18(1-\nu)^2Eh^2k^4}\frac{\Phi_n(\Upsilon_n-\Phi_n)}{\sqrt{\Upsilon_n}}}, \tag{9}$$

where

$$\Upsilon_n = \left(\sqrt{K_J} + \sqrt{K_J+\Phi_n^3}\right)^{2/3}, \tag{10}$$

$$\Phi_n = 2(1-\nu)\left(3n\kappa hk^4\right)^{1/3}, \tag{11}$$

$$K_J = 2[9J_0\check{\epsilon}(1-\nu)]^2(1+\nu)E, \tag{12}$$

and $n \in 1, 2$ is the pattern dimension.

Supplementary Note 4: Crossover Janus Degree

The Janus degree at which the transition between bending and stretching-dominance occurs in clamped-edge, composition → shape programmed monolayers is determined by equating the asymptotic expressions for the two regimes given in Table 1. The results are

$$J_c^{\mathrm{P1}} \approx \left(\frac{9h^4\tilde{\nu}^3}{128E_c r_0^4}\right)^{1/2}, \tag{13}$$

$$J_{\mathrm{c}}^{\mathrm{P2}} \approx \left(\frac{2h^4}{E_{\mathrm{c}}L^3 d}\right)^{1/2}, \tag{14}$$

$$J_{\mathrm{c}}^{\mathrm{P3}} \approx \left(\frac{h^4 k^4 \tilde{\nu}^2}{16E_{\mathrm{c}}}\right)^{1/2}, \tag{15}$$

$$J_{\mathrm{c}}^{\mathrm{P4}} \approx \left(\frac{h^4 k^4 \tilde{\nu}}{16E_{\mathrm{c}}}\right)^{1/2}, \tag{16}$$

$$J_{\mathrm{c}}^{\mathrm{P5}} \approx \left(\frac{h^4}{2\pi^{3/2}E_{\mathrm{c}}\sigma^4}\right)^{1/2}, \tag{17}$$

where superscripts indicate pattern types and $E_{\mathrm{c}} = E_{\epsilon}\tilde{\nu}^3\check{\epsilon}^3/8$.

Supplementary References

[1] J Kang, S Tongay, J Li, and J Wu, "Monolayer semiconducting transition metal dichalcogenide alloys: Stability and band bowing," Journal of Applied Physics **113**, 143703 (2013).

[2] R K Defo, S Fang, S N Shirodkar, G A Tritsaris, A Dimoulas, and E Kaxiras, "Strain dependence of band gaps and exciton energies in pure and mixed transition-metal dichalcogenides," Phys. Rev. B **94**, 155310 (2016).

[3] D Cakır, F M Peeters, and C Sevik, "Mechanical and thermal properties of h-MX_2 (M= Cr, Mo, W; X= O, S, Se, Te) monolayers: A comparative study," Applied Physics Letters **104**, 203110 (2014).

[4] K Lai, W-B Zhang, F Zhou, F Zeng, and B-Y Tang, "Bending rigidity of transition metal dichalcogenide monolayers from first-principles," Journal of Physics D: Applied Physics **49**, 185301 (2016).

[5] J Mann, Q Ma, P M Odenthal, M Isarraraz, D Le, E Preciado, D Barroso, K Yamaguchi, G von Son Palacio, A Nguyen, et al., "2-dimensional transition metal dichalcogenides with tunable direct band gaps: $MoS_{2(1-x)}Se_{2x}$ monolayers," Advanced Materials **26**, 1399–1404 (2014).

[6] A E Maniadaki, G Kopidakis, and I N Remediakis, "Strain engineering of electronic properties of transition metal dichalcogenide monolayers," Solid State Communications **227**, 33–39 (2016).
