# Supplementary material for: The MoSeS dynamic omnigami paradigm for smart shape and composition programmable 2D materials
Source: Nat Commun. 2019 Nov 15;10:5210. doi: 10.1038/s41467-019-12945-5 (PMC6858317; doi:10.1038/s41467-019-12945-5)
Supplement: Supplementary file 2 — Description of Additional Supplementary Files [file 41467_2019_12945_MOESM2_ESM.pdf]

Description of Additional Supplementary Information

The MoSeS Dynamic Omnigami paradigm for smart shape and composition programmable 2D materials

Berry et al.

Supplementary movie 1. Mechanical equilibration of a Janus composition pattern that generates shape P1, starting from a flat state (see Fig. 4a).

Supplementary movie 2. Mechanical equilibration of a Janus composition pattern that generates shape P2, starting from a flat state (see Fig. 4a).

Supplementary movie 3. Mechanical equilibration of a Janus composition pattern that generates shape P3, starting from a flat state (see Fig. 4a).

Supplementary movie 4. Mechanical equilibration of a Janus composition pattern that generates shape P4, starting from a flat state (see Fig. 4a).

Supplementary movie 5. Mechanical equilibration of a Janus composition pattern that generates a Miura-ori geometry, starting from a flat state (see Fig. 4a).

Supplementary movie 6. Actuation of a monolayer containing a circular Janus domain (P1). The direction of an applied electric field of constant magnitude ($|\mathbf{E}_x + \mathbf{E}_y|$) is rotated about the sheet normal (see Fig. 6b).

Supplementary movie 7. Actuation of a monolayer containing a circular Janus domain (P1). The magnitude of an applied electric field aligned with the sheet normal ($\mathbf{E}_z$) is varied through half of a triangle wave period (see Fig. 6b).

Supplementary movie 8. Actuation of a monolayer containing a two-fold asymmetric circular Janus domain. The magnitude of an applied electric field aligned with the plane of the sheet (left to right, $\mathbf{E}_x$) is varied through one triangle wave period (see Fig. 6c).

Supplementary movie 9. Actuation of a monolayer containing a two-fold asymmetric circular Janus domain. The magnitude of an applied electric field aligned with the plane of the sheet (lower left to upper right, $\mathbf{E}_y$) is varied through one triangle wave period (see Fig. 6c).

Supplementary movie 10. Actuation of a monolayer containing a two-fold asymmetric circular Janus domain. The magnitude of an applied electric field aligned with the sheet normal ($\mathbf{E}_z$) is varied through one triangle wave period (see Fig. 6c).

Supplementary movie 11. Actuation of a monolayer containing lamellar Janus domains (P2) of alternating polarity. The magnitude of an applied electric field aligned with the plane of the sheet (left to right, $\mathbf{E}_x$) is varied through one triangle wave period (see Fig. 6d).

Supplementary movie 12. Actuation of a monolayer containing a 2D sinusoidal pattern (P4). The magnitude of an applied electric field aligned with the plane of the sheet (left to right, $\mathbf{E}_x$) is varied through one triangle wave period (see Fig. 6e).

Supplementary movie 13. Actuation of a monolayer containing a 2D sinusoidal pattern (P4). The magnitude of an applied electric field aligned with the plane of the sheet (front to back, $\mathbf{E}_y$) is varied through one triangle wave period (see Fig. 6e).

Supplementary movie 14. Actuation of a monolayer containing a 2D sinusoidal pattern (P4). The magnitude of an applied electric field aligned with the sheet normal ($\mathbf{E}_z$) is varied through one triangle wave period (see Fig. 6e).

Supplementary movie 15. Actuation of a monolayer containing a square lattice of alternating circular Janus domains. The magnitude of an applied electric field aligned with the plane of the sheet (left to right, $\mathbf{E}_x$) is varied through one

triangle wave period (see Fig. 6f).

Supplementary movie 16. Actuation of a monolayer containing a square lattice of alternating circular Janus domains. The magnitude of an applied electric field aligned with the plane of the sheet (front left to back right, $\mathbf{E}_x = \mathbf{E}_y$) is varied through one triangle wave period (see Fig. 6f).

Supplementary movie 17. Actuation of a monolayer containing a square lattice of alternating circular Janus domains. The direction of an applied electric field of constant magnitude ($|\mathbf{E}_x + \mathbf{E}_y|$) is rotated about the sheet normal (see Fig. 6f).

Supplementary movie 18. Actuation of a monolayer containing a square lattice of alternating circular Janus domains. The magnitude of an applied electric field aligned with the sheet normal ($\mathbf{E}_z$) is varied through one triangle wave period (see Fig. 6f).

Supplementary movie 19. Actuation of a monolayer containing concentric Janus ring domains of alternating polarity. The magnitude of an applied electric field aligned with the plane of the sheet (left to right, $\mathbf{E}_x$) is varied through one triangle wave period (see Fig. 6g).

Supplementary movie 20. Actuation of a monolayer containing concentric Janus ring domains of alternating polarity. The direction of an applied electric field of constant magnitude ($|\mathbf{E}_x + \mathbf{E}_y|$) is rotated about the sheet normal (see Fig. 6g).

Supplementary movie 21. Actuation of a monolayer containing concentric Janus ring domains of alternating polarity. The magnitude of an applied electric field aligned with the sheet normal ($\mathbf{E}_z$) is varied through one triangle wave period (see Fig. 6g).

Supplementary movie 22. Actuation of a monolayer containing two-fold asymmetric concentric Janus ring domains of alternating polarity. The magnitude of an applied electric field aligned with the plane of the sheet (left to right, $\mathbf{E}_x$) is varied through one triangle wave period (see Fig. 6h).

Supplementary movie 23. Actuation of a monolayer containing two-fold asymmetric concentric Janus ring domains of alternating polarity. The direction of an applied electric field of constant magnitude ($|\mathbf{E}_x + \mathbf{E}_y|$) is rotated about the sheet normal (see Fig. 6h).

Supplementary movie 24. Actuation of a monolayer containing two-fold asymmetric concentric Janus ring domains of alternating polarity. The magnitude of an applied electric field aligned with the sheet normal ($\mathbf{E}_z$) is varied through one triangle wave period (see Fig. 6h).

Supplementary movie 25. Actuation of a monolayer containing a square lattice of circular Janus domains with randomly chosen polarity. The magnitude of an applied electric field aligned with the plane of the sheet (left to right, $\mathbf{E}_x$) is varied through one triangle wave period (see Fig. 6i).

Supplementary movie 26. Actuation of a monolayer containing a square lattice of two-fold asymmetric circular Janus domains with randomly chosen polarity. The magnitude of an applied electric field aligned with the plane of the sheet (front to back, $\mathbf{E}_y$) is varied through one triangle wave period.

Supplementary movie 27. Actuation of a monolayer containing zigzag Janus domains with alternating polarity on two length scales. The magnitude of an applied electric field aligned with the plane of the sheet (left to right, $\mathbf{E}_x$) is varied through one triangle wave period (see Fig. 6j).
